# Mapping 60 Years of Psychophysiology: A Bibliometric Analysis of Journal Performance, Authorship Trends, and Thematic Evolution

**DOI:** 10.1111/psyp.70002

**Published:** 2025-02-02

**Authors:** Christian Panitz, Carola Dell'Acqua

**Affiliations:** ^1^ Department of Psychology University of Bremen Bremen Germany; ^2^ Department of General Psychology University of Padova Padua Italy

**Keywords:** authorship, bibliometric review, bibliometrix, journal performance, psychophysiology, thematic development

## Abstract

*Psychophysiology*, the flagship journal of psychophysiological research, has played a key role in the field for 60 years. For the present study, we conducted a bibliometric analysis assessing the journal's development in terms of performance, authorship trends, and thematic content for this time span. Over the years, *Psychophysiology* has experienced a consistent increase in manuscript submissions, published articles, and impact factor. Authorship trends showed larger, more diverse author teams, with a growing percentage of female first authors now representing about 50% of submissions and an increase in international collaborations. Thematic content has evolved, shifting from peripheral measures to central nervous system measures like EEG and ERPs while maintaining the journal's long‐standing emphasis on methodological advancements. Research topics have expanded from basic stimulus processing to more complex investigations into emotion, cognition, and psychopathology, with growing interdisciplinary integration. This article provides a quantitative overview of *Psychophysiology*'s contributions and development, aimed at offering insights into the journal's past, current state, and potential future directions in psychophysiological research.

## Abstract 2

This bibliometric review offers a comprehensive quantitative analysis of Psychophysiology's evolution over its 60‐year history, identifying thematic shifts as well as trends in journal performance and authorship. The journal's growth and increasing integration of cognitive and affective research areas highlight the importance of interdisciplinary approaches. We also emphasize the need for greater inclusivity in psychophysiological research, calling for expanded geographic and demographic diversity to enhance the generalizability and global relevance of research in the field.

## 1 Introduction

The year 2023 marked the 60th anniversary of *Psychophysiology*, one of the most established international scientific journals specifically dedicated to the dissemination of cutting‐edge psychophysiological research. The journal was founded in response to the need to bring together researchers who sought to explore the relationship between the mind and the body, as before that time, according to the opening of the inaugural issue, “*scientists had to search through more than 80 Journals to find reports of psychophysiological research*” (Ax 1964). The 1960s, with the formation of the Society for Psychophysiological Research (SPR) in 1960 and of *Psychophysiology* in 1964, set the basis for the establishment of psychophysiological research as a contemporary and independent discipline (Holguín and Cadaveira 2002). Since then, *Psychophysiology* has been publishing research aimed at building and improving theoretical models on the bidirectional associations between social, psychological, and behavioral phenomena and central and peripheral physiological functions, both in healthy and clinical populations (e.g., with psychosomatic or psychiatric disorders; Cacioppo and Tassinary 1990). Over the years, the journal has maintained a close and enduring relationship with the SPR. This connection is reflected in the alignment of research topics featured in the journal and those presented at the Society's annual meetings, as well as the overlap between the Editorial Board members and the SPR Board members, SPR fellows, and the recipients of notable SPR honors (e.g., Distinguished Contributions to Psychophysiology Awards). Notably, Early Career Award winners are encouraged to submit a paper to the journal based on their award address at the annual meeting, highlighting the reciprocal influence between Psychophysiology and the Society. This strong collaboration with SPR has helped Psychophysiology shape and promote the growth of contemporary psychophysiological research over the past six decades. Particularly, *Psychophysiology* has shaped and promoted the growth of contemporary psychophysiological research and set methodological standards with guideline papers (e.g., Blumenthal et al. 2005; Keil et al. 2022; Picton et al. 2000; Steinhauer et al. 2022), and continues to be an important outlet for psychophysiologists to disseminate methodologically rigorous work and investigate psychophysiological mechanisms.

Although current research relies upon theoretical frameworks advanced decades ago, the past sixty years have been characterized by an ever‐expanding progression in the employed methodological tools and technology. For instance, the field has encountered a shift from analog systems to sophisticated digital systems that enable the exploration of physiological measures with unparalleled precision (e.g., impedance cardiography, high‐density EEG, functional magnetic resonance imaging). These technological developments have led to a further shift from the study of peripheral, visceral functions to central measures. Indeed, much of the early research published in *Psychophysiology* focused on peripheral components (e.g., autonomic responses) and lower reflexes (e.g., baroreceptors) associated with basic psychological processes (e.g., conditioning; Berntson, Cacioppo, and Sarter 2002). In contrast, recent research is predominantly centered on the study of the human brain (both in health and pathology), to the extent that, almost a decade ago, editor‐in‐chief Monica Fabiani devoted an editorial entitled “The embodied brain” to encourage scientists to “re‐embody” the brain, that is, to integrate multiple bodily systems to fully characterize the behaving human organism (Fabiani 2015).

With *Psychophysiology*'s recent anniversary, this moment provides an opportunity to both highlight the productivity and impact of psychophysiological research, but also to objectively and critically examine the journal's development over time. To this end, the current study employed bibliometric analyses to portray and analyze the entire publication history (1964–2023) of *Psychophysiology*. Bibliometric analyses are methods that use quantitative tools to map and analyze large sets of academic literature, allowing researchers to track the evolution of a field, identify influential works, and reveal emerging trends (Zupic and Čater 2015). For the present study, we chose analyses that aim at providing objective measures of productivity, authorship, collaboration, citation patterns, and keyword patterns. We thereby aimed to gather insights into how psychophysiological knowledge has been produced and disseminated and how *Psychophysiology* as a scientific journal has evolved. More specifically, we aimed to analyze (i) *Psychophysiology*'s performance over time, using standard indicators such as the number of publications and submissions as well as the impact factor; (ii) authorship changes, analyzing factors such as the number of co‐authors, gender of first/corresponding authors, and geographical distribution; (iii) influential, seminal papers, investigating citation patterns between highly cited publications; and (iv) the thematic development, examining how key terms and key term patterns have emerged and evolved over the past 60 years. Particularly, a science mapping approach was used (van Raan 2014) to trace the thematic development, analyze, and visualize occurrences and co‐occurrences of key terms, and create a historic map of seminal *Psychophysiology* publications revealing thematic clusters and citation patterns between papers. By mapping these indicators over time, we aimed to provide a structured overview of the journal's current state, its contributing authors, and the thematic landscape of psychophysiological research—illustrating the major developments and considering potential future directions of the field.

## 2 Method

### 2.1 Data

#### 2.1.1 Literature Search

We searched Web of Science (WoS) on September 6, 2024, to acquire bibliometric data for *Psychophysiology* documents from 1964 to 2023. We included publications of type “Article” and “Review Article” while excluding meeting abstracts, editorial material, notes, book reviews, letters, biographical items, items about individuals, discussions, meeting info, and a software review. We also explicitly excluded “Early Access” papers, as they had not yet been assigned to an issue, and “Correction” papers. The Boolean search term is presented in Table 1. Additionally, search results were refined using the GUI, and the years were to indicate the *final* publication years.

**TABLE 1:** Boolean search terms for Web of Science search.

SO = ((“Psychophysiology”))
AND
PY = ((1964–2023))
AND
DT = ((Article) OR (Review))
NOT
DT = ((Correction) OR (Early Access))

Abbreviations: DT, document type; PY, publication year; SO, journal name.

The full bibliometric record and the cited references were downloaded for each document. Data preparation and analysis were conducted in R 4.3.1 (R Core Team 2023) and in the RStudio environment 2023.06.0 (Posit Team 2023) using the *bibliometrix* package 4.1.3 (Aria and Cuccurullo 2017).

Bibliometric data for a total of 5869 publications were retrieved. Article titles for science mapping were available for all publications. Complete data for the countries of the authors' research institutions was available from 1998 to 2023 and was used for statistical analyses. On the basis of this dataset, we conducted analyses on publication numbers, author teams (team size and countries of research institutions), seminal papers (see *historical direct citation network*), as well as occurrence and co‐occurrence analyses of key terms extracted from publication titles.

#### 2.1.2 Additional Data Sources

We obtained 2‐year impact factors for the years 1997 to 2021 from the *Clarivate Journal Citations Report* (retrieved on March 22, 2024). In addition, numbers of annual submissions to the journal were obtained from *Wiley Journal Insights* (2020–2023; retrieved on August 9, 2024) and the Editors‐in‐Chief of *Psychophysiology* (1980–2019). Finally, annual percentages of first or corresponding authors' gender (male vs. female) were provided by the Editors‐in‐Chief for the years 1994–2023, with missing data for the years 2005 and 2007 to 2010. The change to Manuscript Central as a submission system occurred on January 1, 2006, resulting in first‐author gender being recorded prior to and corresponding‐author gender thereafter. Due to the extreme overlap between first and corresponding authors, the data were pooled and treated as a single category for analysis.

#### 2.1.3 Preprocessing of Title Key Terms

For content analyses, key terms were extracted from the article titles. To this end, in the first step, we defined key terms consisting of multiple words (e.g., “heart rate”). Recurring word combinations of 2 to 4 words were identified by the *tableTag* function in *bibliometrix* and defined as a single key term if they had at least 5 occurrences across publications. Words were grouped in a way that longer combinations were prioritized over shorter combinations (i.e., “heart rate variability” was not split up into “heart rate” and “variability”). Afterward, all key terms identified by the *tableTag* function (key term lengths: 1 to 4 words) were exported for manual screening, after filtering out stop words commonly used in the English language (e.g., “a,” “the,” “is,” “are”) that were listed in the *stop_words* list of the *tidytext* package (Silge and Robinson 2016) for 1‐word terms and in the *wordstop* list of the *bibliometrix* package for 2‐word terms.

The exported key terms were screened independently by the two authors to create a key term dictionary. This was done for two purposes: First, uninformative key terms (e.g., “study” or “participants”) were deleted from the list of terms to analyze. Second, key terms were recoded if several key terms were deemed to have essentially the same meaning in the context of this review (e.g., “ERP,” “event‐related potential,” and “event‐related potentials”). Disagreements were resolved by discussion. We share the list of all extracted terms and the dictionary on OSF (https://osf.io/6t4u5/).

### 2.2 Data Analysis

#### 2.2.1 Journal Performance

Journal performance over the years was assessed using (a) the number of annual publications, (b) the number of annual submissions to the journal, and (c) the 2‐year impact factor. Annual counts of publications were obtained using the *biblioanalysis* function of the *bibliometrix* package on the bibliometric dataset.

#### 2.2.2 Author Team Characteristics

Characteristics of author teams included (a) the number of co‐authors, (b) the gender of the first/corresponding author, and (c) the countries of the authors' affiliated research institutions. The number of co‐authors was extracted from the bibliometric dataset for all years, and annual means and medians were compared. For gender data, we reported the annual percentage of female first/corresponding authors from 1994 to 2023. Finally, country data was analyzed for the years with a complete record, that is, 1998 to 2023, and for the 15 countries with the most publications in this time range. For each year, we computed the accumulated number of publications (since 1998) by country and the percentage of publications with authors from research institutions in at least two different countries. In addition, we mapped the number of international co‐authorships for the top 15 publishing countries in a network plot.

#### 2.2.3 Science Mapping

##### 2.2.3.1 Historical Direct Citation Network

To obtain an overview of seminal papers in *Psychophysiology*, we constructed a historical direct citation network (Garfield 2004) as implemented in the *histNetwork* and *histPlot* functions of the *bibliometrix* package. Fifty papers with the highest local citation counts (i.e., publications that have been cited by other *Psychophysiology* publications) were extracted, and a network was constructed in which nodes represent papers and are linked if one paper cites the other. In addition, papers were clustered by the *infomap* algorithm as implemented in the *cluster_infomap* function of the *igraph* package (Csardi and Nepusz 2006). The algorithm defines clusters to minimize the expected description length of a random walker trajectory through the network (expressed as Shannon entropy)—maximizing information flow within clusters and minimizing flow between clusters (see Rosvall and Bergstrom 2008, for details).

##### 2.2.3.2 Key Term Development

The most frequently used key terms—extracted from the publication titles—were identified for the entire body of publications and separately for each of the six decades of *Psychophysiology*. Additionally, the accumulated count of the top 10 key terms of all time were plotted over years.

##### 2.2.3.3 Key Term Co‐Occurrences

Relationships between key terms were assessed via co‐occurrence networks, separately for the time windows 1964–1983, 1984–2003, and 2004–2023 (see Figures S3–S14 and Tables S5–S10 for analyses by decade). To this end, occurrences and co‐occurrences of the top 50 frequent key terms in the respective time window were counted using *bibliometrix* functions. In order to visualize the resulting network and group key terms into clusters, co‐occurrence networks were created using the *cocMatrix* function from the *bibliometrix* package and custom code. Exported data was then imported in VOS Viewer 1.6.19 (van Eck and Waltman 2010).

VOS Viewer displays each key term as a node in a two‐dimensional network, where nodes are connected based on co‐occurrence with other key terms. The strength of a link between two nodes, indicated by the line width, reflects the absolute number of times two key terms co‐occur. The size of a node represents the total strength of its links, which is the sum of all co‐occurrences of that key term with others in the network. The spatial proximity of nodes indicates the associative strength between terms, which is a normalized measure of co‐occurrence. In VOS Viewer, the associative strength between two nodes *i* and *j*, is calculated as $s_{\mathrm{ij}}=\frac{2ma_{\mathrm{ij}}}{k_{i}k_{j}}$ where *k* represents the total link strength of a node, m is the total link strength in the network, and *a*_*ij*_ is the absolute number of co‐occurrences of the two key terms (van Eck and Waltman 2009, 2014).

VOS Viewer generates a map by minimizing the sum of Euclidean distances between nodes in two‐dimensional space, weighted by their associative strength (van Eck et al. 2010; van Eck and Waltman 2014). Key terms are then assigned to clusters by maximizing the function $V\left(c_{1},\ldots ,c_{n}\right)=\sum_{i<j}\delta \left(c_{i},c_{j}\right)\left(s_{\mathrm{ij}}-\gamma \right)$. Here *c*_*i*_ denotes the cluster assigned to the *i*th node, and $\delta \left(c_{i},c_{j}\right)$ denotes a function that returns 1 if *c*_*i*_ *== c*_*j*_ (i.e., if two key terms are assigned to the same cluster) and 0 otherwise (van Eck and Waltman 2014). This function is multiplied by the difference between the nodes' associative strength (*s*_*ij*_) and a resolution parameter *γ*. Lower values for *γ* will result in fewer and larger clusters, with *γ* = 0 producing one single cluster. For the current analyses, we used VOS Viewer's default of *γ* = 1. In addition, we set the minimum cluster size to 3, smaller clusters were merged with others.

To quantitatively describe the clusters, we computed centrality and density measures, as proposed by Callon, Courtial, and Laville (1991) and outlined by Cobo and colleagues (2011). Callon's centrality measures a cluster's relevance to the entire field by evaluating its connections to nodes in other clusters. It is defined as $C=10\sum s_{\mathrm{kh}}$, where *s* represents the associative strength between node *k* in a given cluster and node *h* in a different cluster. Callon's density, which indicates how developed a cluster is based on internal connectivity, is calculated as $D=100\left(\sum s_{\mathrm{ij}}/w\right)$ where *s*_*ij*_ is the associative strength between two nodes within the same cluster, and *w* is the number of nodes in the cluster. Note that Cobo and colleagues use the equivalence index, a different but related quantification of normalized co‐occurrence data. Here, we computed Callon's centrality and density based on associative strength as calculated by VOS Viewer.

To visualize clusters' centrality and density values in comparison, we created thematic maps in which each cluster is plotted in 2‐D space as a circle, with its size proportional to the total occurrences of all keywords within the cluster. The *x*‐axis represents Callon's centrality (indicating the extent to which a cluster is connected to other clusters in the network) while the *y*‐axis represents Callon's density (reflecting the internal coherence and development of the cluster). As our centrality and density measures are influenced by cluster size, total network size, and overall keyword distribution, the resulting scores are relative and should not be interpreted in absolute terms. To assist interpretation, the maps include dashed lines corresponding to the unweighted arithmetic means of centrality and density across all clusters. These lines divide the map into four quadrants, reflecting a general categorization framework proposed by Callon, Courtial, and Laville (1991): *Emerging* or *declining* themes have low centrality and density, reflecting underdeveloped areas of limited relevance. *Specialized* or *niche* themes have high density but low centrality, indicating strong internal connectivity but relative isolation. *Basic* or *transversal* themes have high centrality and low density, meaning they connect different clusters but lack strong internal links. Finally, *motor* themes exhibit high levels of both centrality and density, representing well‐developed subfields that structure the broader research area. Importantly, while clusters with higher centrality may be considered more integral to the broader field, and those with higher density may be seen as more internally coherent, their positioning should be interpreted in relative terms and not as an absolute measure of importance or quality.

## 3 Results

### 3.1 Journal Performance

**FIGURE 1:**
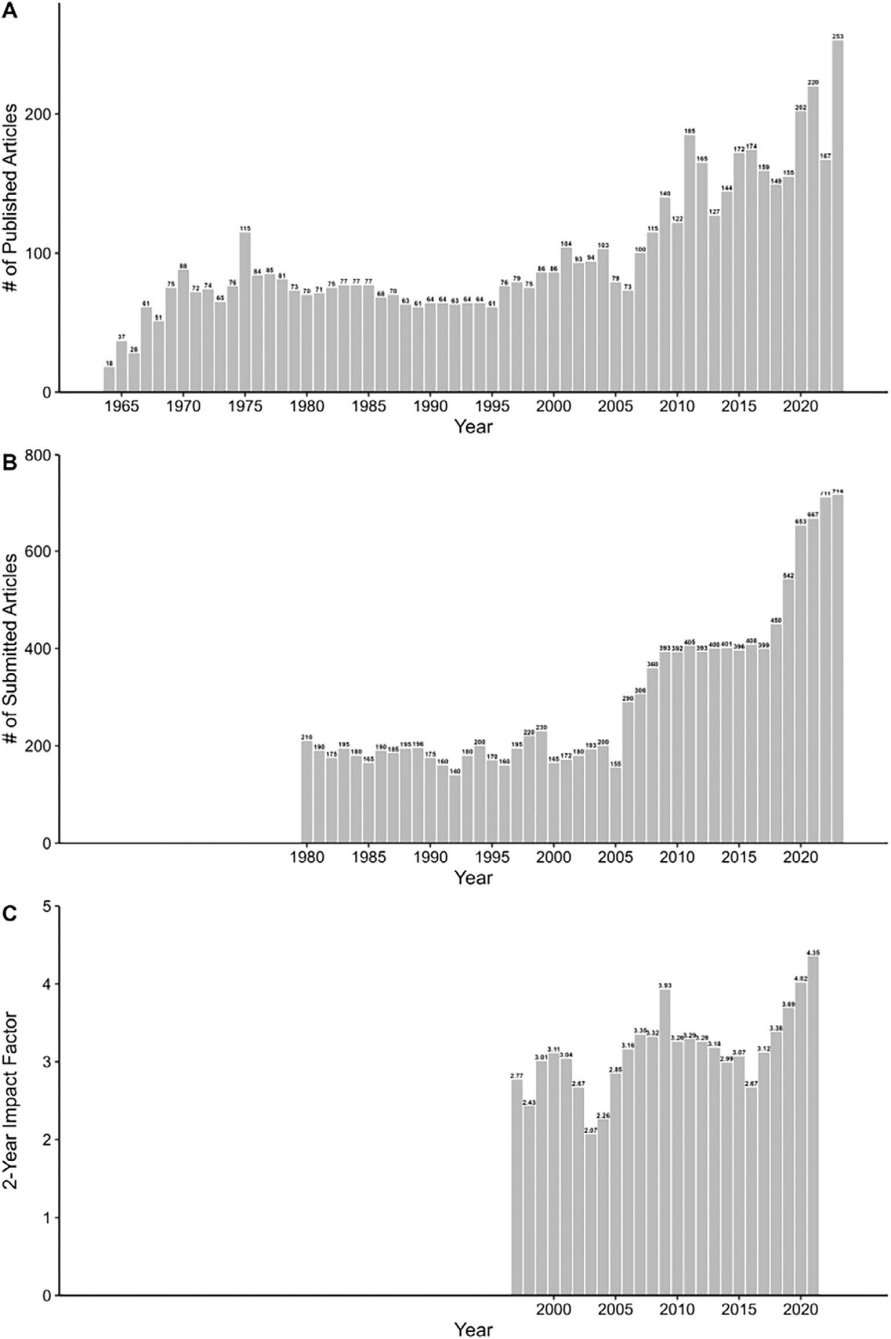
(A) Number of publications from 1964 to 2023. (B) Number of submissions from 1980 to 2023. (C) 2‐year impact factor from 1997 to 2021.

The number of annual publications in *Psychophysiology* increased from 37 to 167 in the years from 1964 to 2023, with a peak of 253 articles in 2023 (Figure 1A). Numbers of annual submissions were stable and in the upper one‐hundreds in the years from 1980 to 2005. From 2006 to 2009, there was a marked and sustained increase in submissions to around 400 submissions annually. Finally, from 2018 to 2023 there was another surge, with recent numbers over 700 submissions (Figure 1B). In the 25 years we could obtain the 2‐year impact factor for (1997 to 2021), the impact factor mostly varied around 3 (median: 3.12, 1st quartile: 2.85, 3rd quartile: 3.32) with its lowest value in 2003 (impact factor: 2.07) and its highest value recently, in 2021 (4.35). The impact factor has been increasing since 2017 (Figure 1C).

### 3.2 Author Teams

**FIGURE 2:**
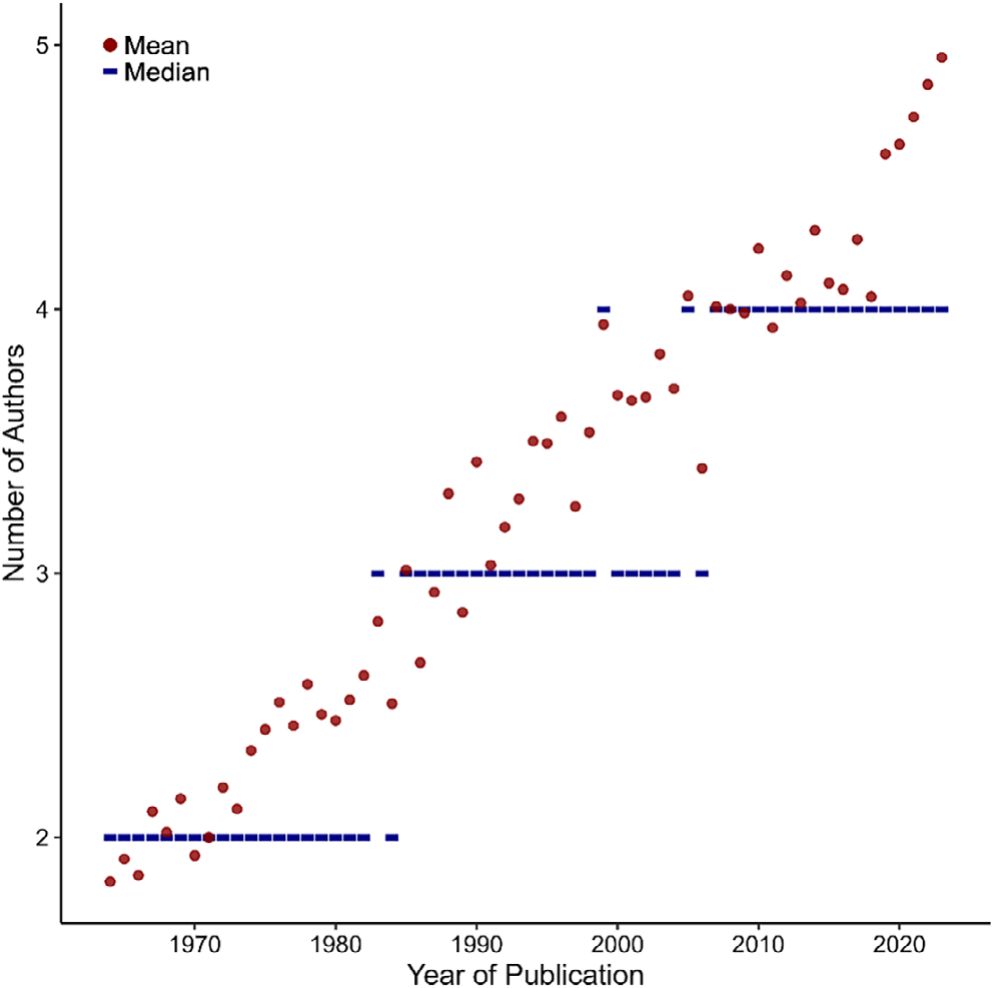
Mean (red dots) and median (blue bars) number of authors per publication by publication year. Figure S1 also shows the distribution of team size across publications and years.

The average size of author teams has been steadily increasing from *M* = 1.83 in 1964 to *M* = 4.95 in 2023 (Figure 2; see Table S1 for numbers by year). On the one hand, this was due to an increase in publications with large author teams (also see Figure S1 for the distribution of author team sizes across publications). At the same time, there was a relative decrease of small author teams, and especially single‐authored papers. Accordingly, median team size increased from 2 to 3 in the mid‐1980s and to 4 in the late 2000s.

**FIGURE 3:**
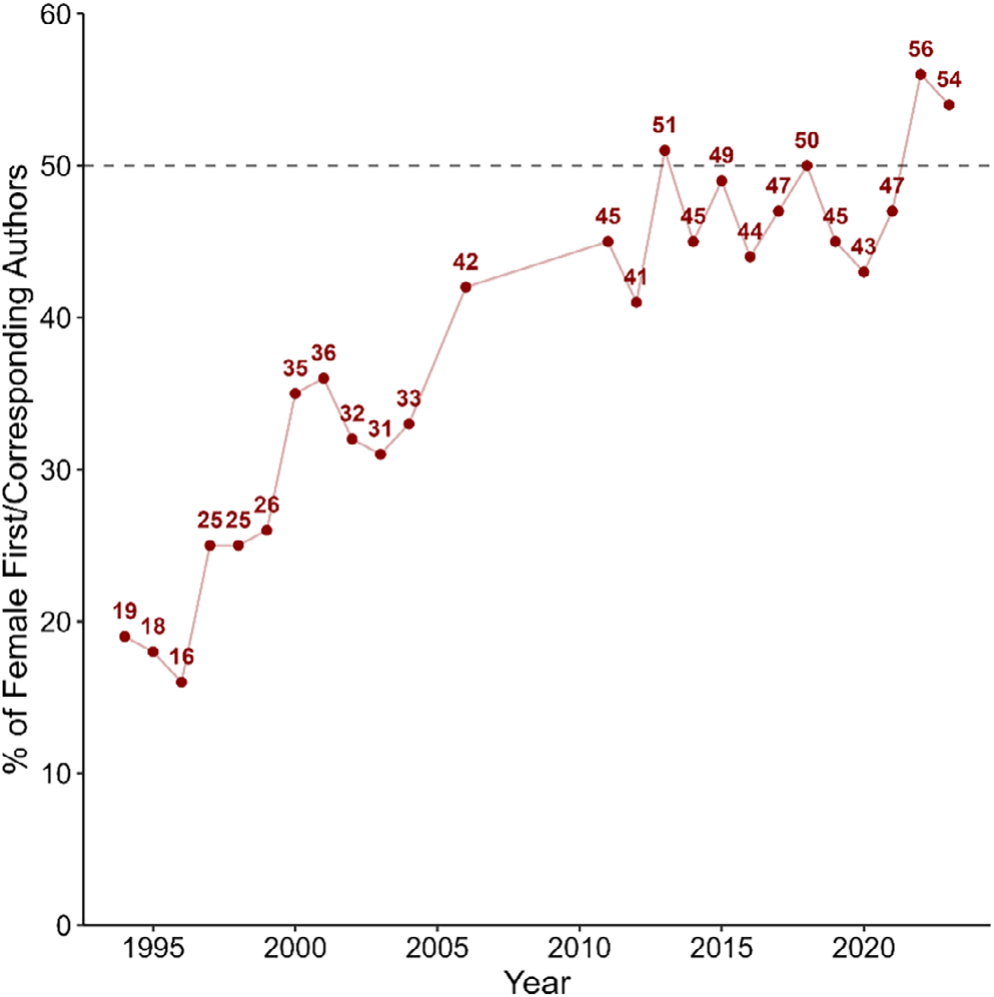
Percentage of female first/corresponding authors (gender) by publication year.

With regard to gender, the percentage of female first/corresponding authors in *Psychophysiology* publications was still below 20% in the mid‐1990s (Figure 3). From there on, the percentage increased steadily until around 2011 (45%). From 2011 to 2023, the percentage of female first/corresponding authors has varied mostly between 40% and 50% (*Md* = 47), reaching or surpassing the 50% mark only in 2013 (51%), 2018 (50%), 2022 (56%), and 2023 (54%).

With regard to countries of authors' research institutions, the 15 most publishing countries were mostly from Europe and North America, with the USA (most publications: 3710) and Germany (2nd most: 1273) leading. Non‐European/American countries included China (3rd most: 785), Australia (7th: 406), Japan (13th: 151), and Israel (15th: 123; see Table 2 for top 15 publishing countries and Table S2 for a list of all publishing countries since 1998). Note that these are the accumulated numbers from 1998 to 2023 (only years with complete country data) and that one publication can count toward multiple countries, depending on co‐authorships.

**TABLE 2:** Accumulated publication numbers for countries of authors' research institutions from 1998 to 2023.

Country	Publications	Country	Publications	Country	Publications
USA	3710	Canada	437	Switzerland	208
Germany	1273	Australia	406	Belgium	153
China	785	Spain	283	Japan	151
UK	591	France	244	Finland	146
Netherlands	490	Italy	238	Israel	123

**FIGURE 4:**
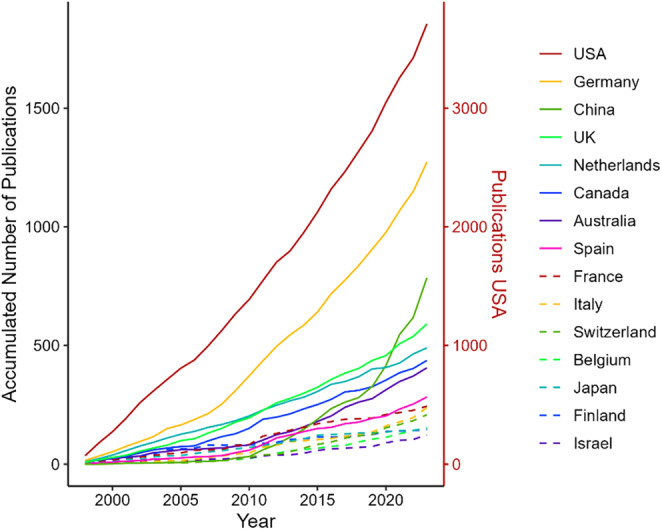
Accumulated publication numbers of the top 15 publishing countries of authors' research institutions from 1998 to 2023.

Inspection of the development over time revealed that authors from research institutions in the United States have constantly been publishing the most articles in each year (Figure 4). While German research institutions held the second position for most of the time, publications from Chinese institutions have been increasing steeply since 2019, resulting in the second‐highest numbers in three out of the last four years. In addition, publications from Italian and Spanish institutions have increased significantly in the last 5 years.

**FIGURE 5:**
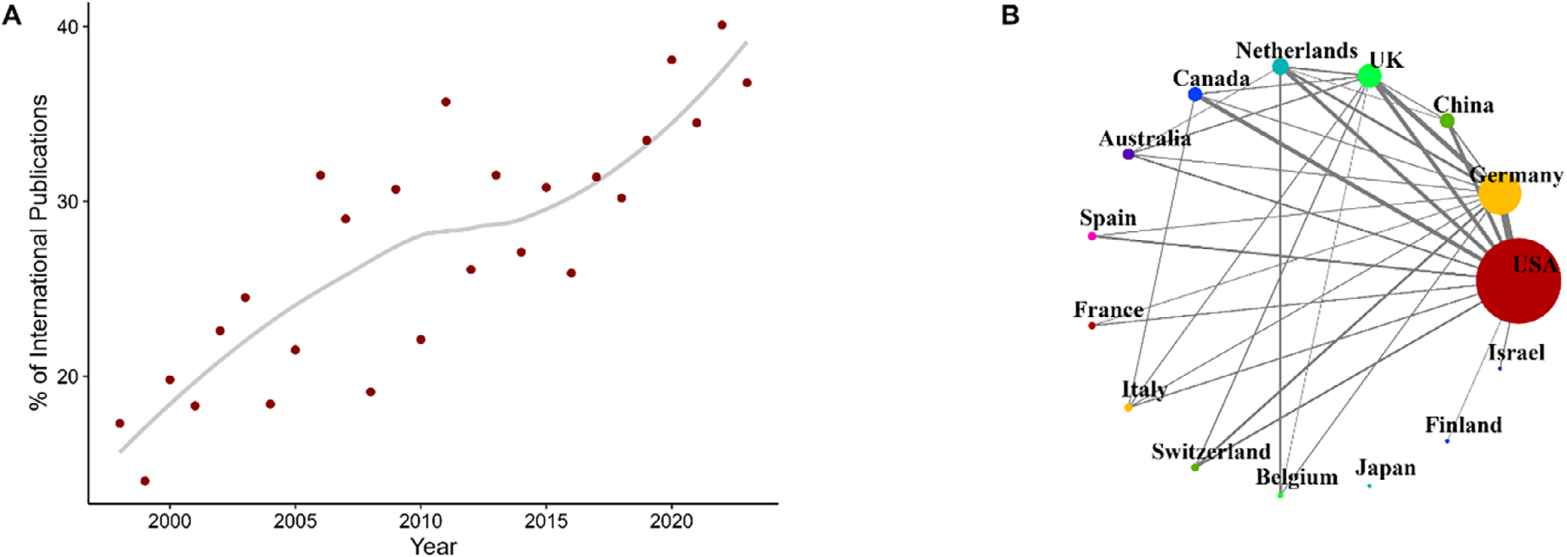
Internationally co‐authored publications in *Psychophysiology* from 1998 to 2023. (A) Percentage of internationally co‐authored publications relative to all publications by year. Regression line fitted using LOESS. (B) Overview of publications with authors from multiple countries. Countries are ordered counter‐clockwise by the total number of publications from highest (USA) to lowest (Israel). Circle size reflects the number of publications co‐authored with at least one other top‐15 country. Line width scales with the number of publications with at least one author from both countries. Lines are only plotted for pairs of countries with at least 10 co‐authored publications.

Meanwhile, the percentage of publications with authors from research institutions in at least two different countries has steadily increased from around 15% in the late 1990s to around 40% in recent years (Figure 5A). The number of internationally co‐authored publications was strongly correlated with the total publication output (Figure 5B; also see Table S3 for numbers of co‐authored papers between countries). Notably, Chinese institutions published less with international collaborators relative to their total publication output. Similarly, Japanese institutions did not publish at least 10 publications with any other country. Meanwhile, Italy and Switzerland showed higher numbers of collaboration relative to their total output.

### 3.3 Science Mapping—Historical Direct Citation Network

**FIGURE 6:**
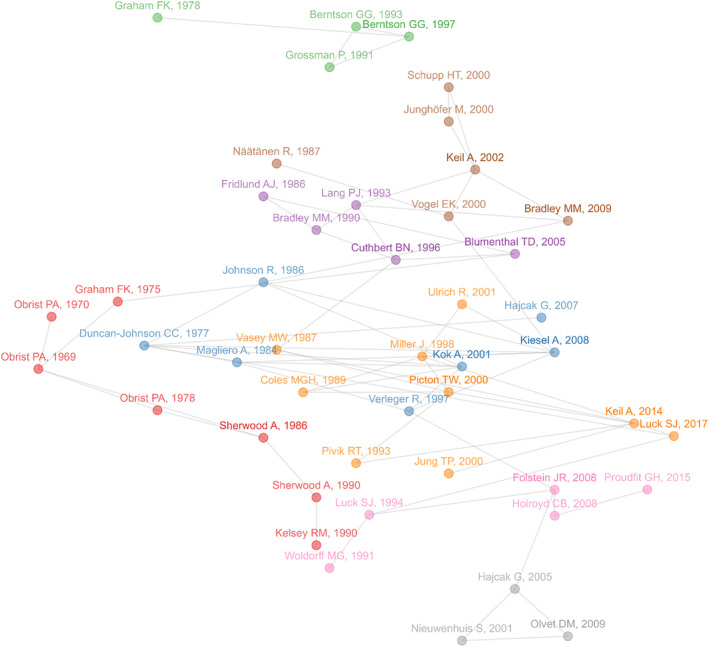
Historical Direct Citation Network. Nodes represent the locally most cited publications as of September 6, 2024. Publications are linked to other publications they cited. The colors represent the eight clusters linking papers: Cardiovascular psychophysiology (red), P300 and prediction errors (blue), EMG methods & EMG affective picture viewing (violet), respiratory sinus arrhythmia & heart‐rate variability (green), EEG methods & guidelines (orange), and error‐related brain activity (gray), the heterogenous cluster of ERP studies on cognitive control, visual search, and feedback processing (pink), and, the heterogenous cluster of studies on the N1 ERP component, artifact control in high‐density EEG, and affective picture processing & attention (brown).

From the fifty selected papers with the highest local citation counts, 46 had links between each other (i.e., cited at least one or were cited by at least one of the other selected papers) and were therefore included in the network (Figure 6). The *infomap* algorithm identified 8 clusters linking publications on cardiovascular psychophysiology (red), P300 and prediction errors (blue), EMG methods & EMG in affective picture viewing (violet), respiratory sinus arrhythmia & heart‐rate variability (green), EEG methods & guidelines (orange), and error‐related brain activity (gray). There were also two more heterogeneous clusters: while the pink cluster contained ERP studies on cognitive control, visual search, and feedback processing, the brown cluster contained papers on the N1 ERP component, artifact control in high‐density EEG, and affective picture processing & attention. A complete list of the papers included in the network can be found in the Table S4.

### 3.4 Science Mapping—Key Term Development

**FIGURE 7:**
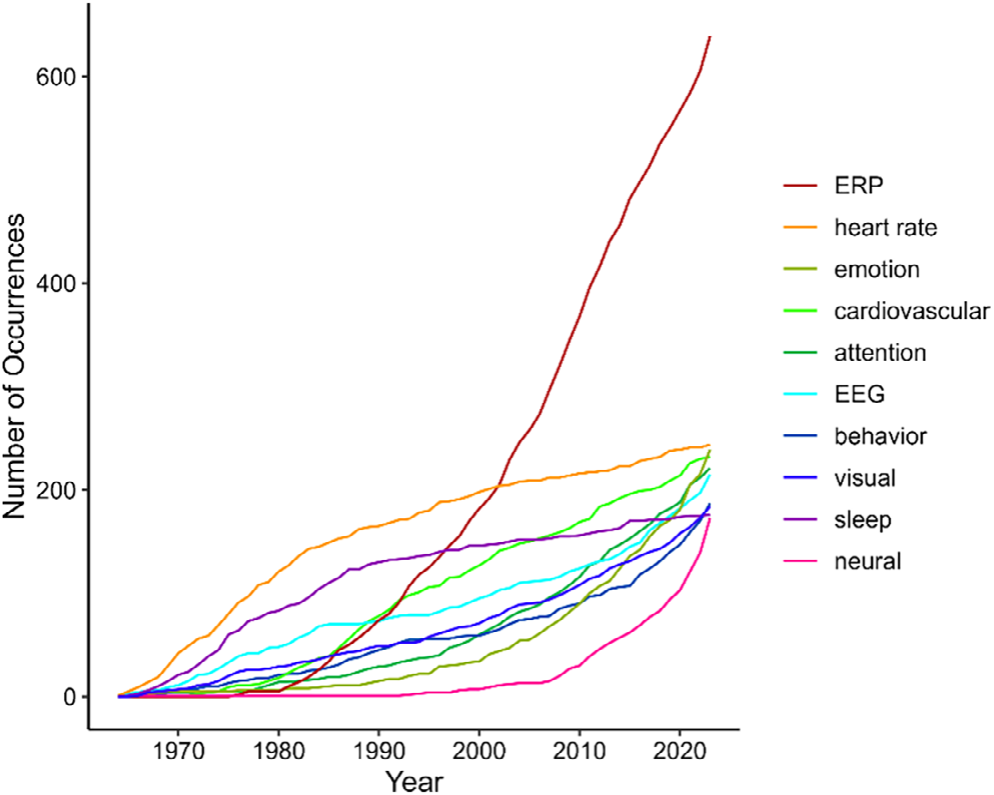
Accumulated number of occurrences of all‐time top 10 key terms from 1964 to 2023.

In order to track thematic development, we extracted the 10 most common key terms appearing in article titles for each of the journal's decades. In the first 10 years, papers in *Psychophysiology* mostly addressed the topics of sleep, conditioning, and orienting while using mostly peripheral autonomic measures (Table 3). Peripheral measures (heart rate, cardiac, and electrodermal) were predominantly mentioned in the first two decades but have progressively made way for EEG and ERPs. Indeed, although the term “EEG” was already a top‐10 key term in early journal titles, EEG studies became more popular over time until “ERP” became the top keyword in the journal's third decade (i.e., 1984–1993) and continued to be so until 2023. The P300 was the most commonly mentioned component, particularly in the time windows from 1984 to 2013. Of note, key terms from the last decade (2014–2023) do not include any peripheral measure. Overall, psychological constructs mentioned in article titles have changed over time. Initially, researchers focused on topics such as “orienting,” “conditioning,” and “habituation,” but as time progressed, they broadened to “attention” (emerging between 1984–1993), “emotion” (1994–2003), and “cognition” (2004–2013), which have consistently ranked among the top 10 key terms up to the present day. Table 3 lists the top 10 key terms across the time windows and for all time. In addition, Figure 7 shows the time course of accumulated occurrences for the top 10 key terms over the years. Key term trends over time for the top 50 key terms are depicted in Figure S2.

**TABLE 3:** Most frequent key terms in article titles over time.

1964–1973	1974–1983	1984–1993	1994–2003	2004–2013	2014–2023	All time
heart rate	heart rate	ERP	ERP	ERP	ERP	ERP
SCR	sleep	cardiovascular	cardiovascular	attention	emotion	heart rate
sleep	EEG	sleep	attention	emotion	neural	emotion
conditioning	feedback	P300	P300	electro‐physiological	behavior	cardiovascular
autonomic	function	behavior	human	auditory	EEG	attention
cardiac	electrodermal	heart rate	heart rate	perception	attention	EEG
EEG	autonomic	blood pressure	memory	cardiovascular	electro‐physiological	behavior
reaction time	cardiac	stress	visual	P300	adult	visual
human	conditioning	human	scene	neural	memory	sleep
eye movement	habituation	stimulus	auditory	visual	perception	neural

*Note:* Top 10 key terms for each decade and for all time in descending order of frequency.

### 3.5 Science Mapping—Key Term Co‐Occurrences

**FIGURE 8:**
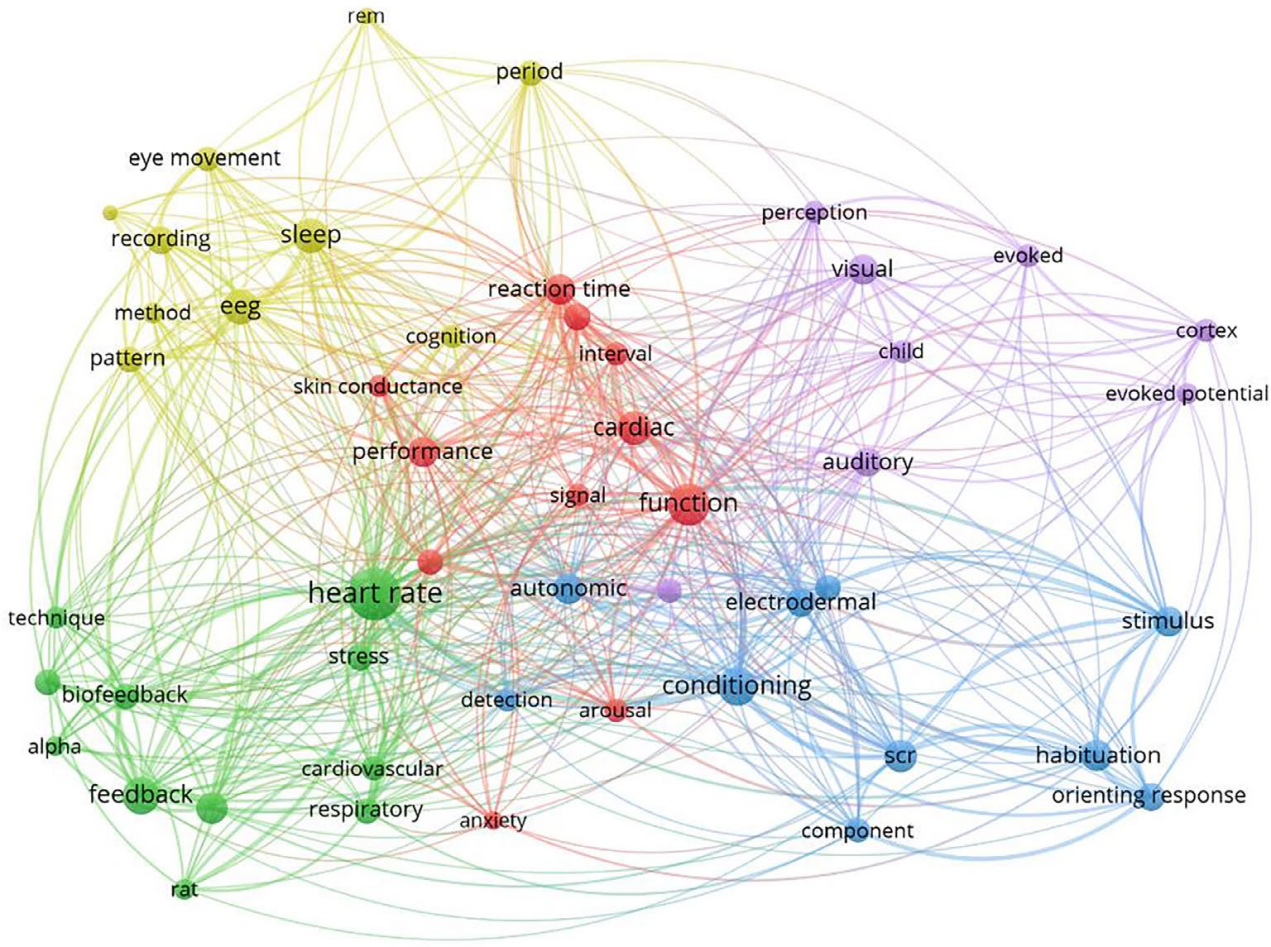
Co‐occurrence network for key terms extracted from titles of publications from 1964 to 1983.

**FIGURE 9:**
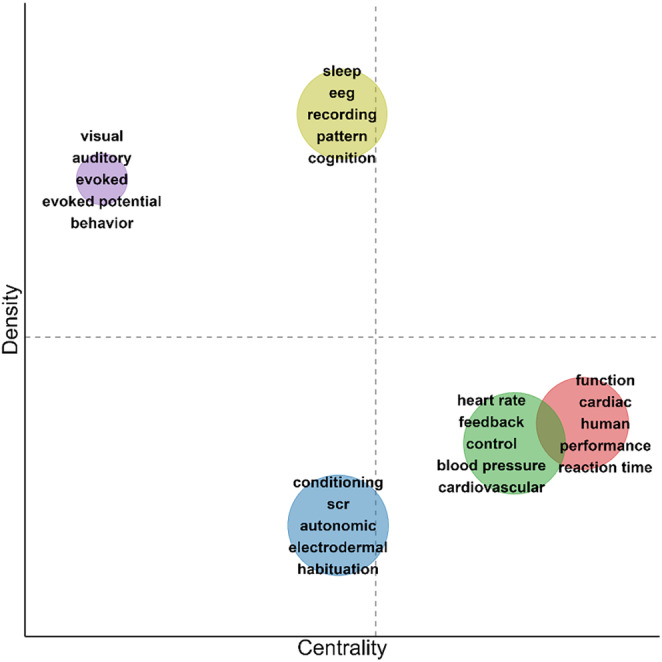
Thematic map for key term clusters from 1964 to 1983. Each cluster is depicted as a circle with its size reflecting the total occurrences across all key terms in the cluster and its position indicating Callon's centrality (*x*‐axis) and Callon's density (*y*‐axis). Dashed lines indicate unweighted average centrality and density across all clusters. For each cluster, the 5 most frequent keywords are listed.

For the time window from 1964 to 1983, 5 co‐occurrence clusters were identified (Figure 8, Table 4), that were of similar size (8 to 11 key terms). Clusters 1 (red) and 2 (green) had higher values in centrality in comparison but lower values in density (Figure 9). Cluster 1 consisted of a heterogeneous set of key terms, for example, outcome variables (*cardiac, performance, reaction time, skin conductance*) and shared borders with all other clusters, suggesting that this cluster represents widely used outcome measures and constructs. Meanwhile, Cluster 2 included a cardiovascular theme (*heart rate, blood pressure, cardiovascular*) together with *feedback, biofeedback*, and other terms that were situated further marginally. The cluster's high centrality is likely boosted by *heart rate*, which is the most occurring term in the first 20 years of *Psychophysiology* and is situated centrally, connecting Cluster 2 to other clusters. Meanwhile, Clusters 4 (yellow) and 5 (purple) had the highest values in density but less so in centrality, suggesting that these were more specialized subfields. Cluster 4 appeared to reflect sleep research (*sleep, rem, eye movement*) using EEG. Cluster 5 was centered around cortical potentials (*cortex, evoked, evoked potential*) in the perception of different kinds of stimuli (*perception, visual, auditory*). Finally, Cluster 3 (blue) had low levels of centrality and density. It included research on electrodermal (*electrodermal, scr*, also *autonomic*) measurement of basic stimulus processing and learning (*detection, stimulus, conditioning, habituation, orienting response*). While terms like *conditioning* and *electrodermal* connected Cluster 3 to other clusters, *habituation*, *orienting response*, and *stimulus* were rather specific for this cluster.

**TABLE 4:** Key terms and density for clusters of key term co‐occurrence network—1964 to 1983.

Cluster	Key terms	Size	Centrality	Density
1	function, cardiac, human, performance, reaction time, level, skin conductance, arousal, interval, signal, anxiety	11	1965 (1)	1774 (3)
2	heart rate, feedback, control, blood pressure, cardiovascular, biofeedback, respiratory, stress, rat, technique, alpha	11	1860 (2)	1743 (4)
3	conditioning, scr, autonomic, electrodermal, habituation, stimulus, orienting response, detection, component, differential	10	1589 (4)	1616 (5)
4	sleep, eeg, recording, pattern, cognition, rem, eye movement, method, period, skin resistance	10	1595 (3)	2252 (1)
5	visual, auditory, evoked, evoked potential, behavior, child, perception, cortex	8	1225 (5)	2151 (2)

*Note:* Key terms: key terms included in the cluster, sorted for occurrences. Size: number of key terms in the cluster. Centrality: Callon's centrality as specified by Cobo et al. (2011) with cluster rank in parentheses. Density: Callon's density as specified by Cobo et al. (2011) with cluster rank in parentheses.

Abbreviations: eeg, electroencephalography; rem, rapid eye movement; scr, skin conductance response.

**FIGURE 10:**
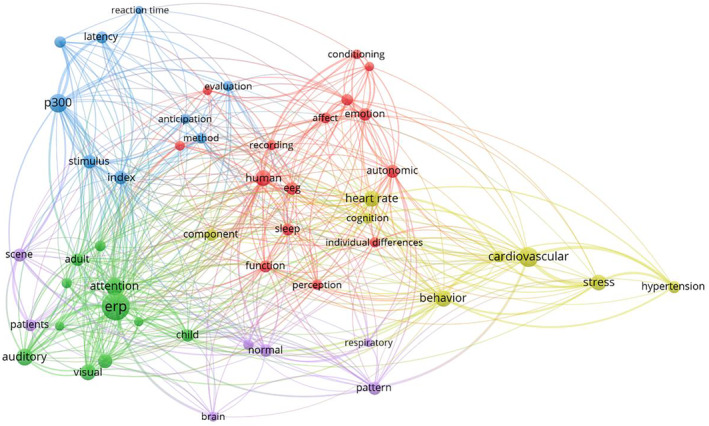
Co‐occurrence network for key terms extracted from titles of publications from 1984 to 2003.

**FIGURE 11:**
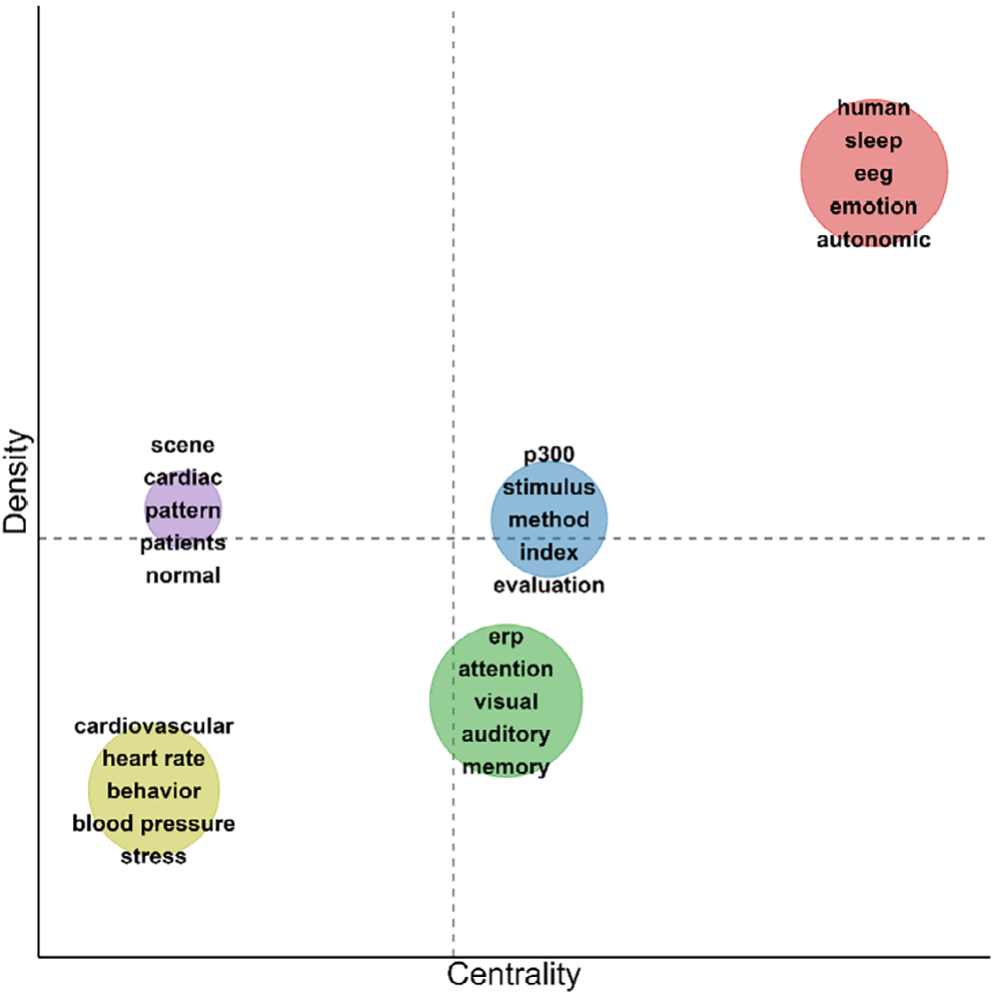
Thematic map for key term clusters from 1984 to 2003. Each cluster is depicted as a circle with its size reflecting the total occurrences across all key terms in the cluster and its position indicating Callon's centrality (*x* axis) and Callon's density (*y* axis). Dashed lines indicate unweighted average centrality and density across all clusters. For each cluster, the 5 most frequent keywords are listed.

For the time window from 1984 to 2003, again, 5 clusters were identified (Figure 10, Table 5), although cluster sizes varied more strongly (7 to 15 key terms). Notably, Cluster 1 (red) had the highest centrality and density by far and was also the largest cluster (Figure 11). It included a variety of research contexts (*sleep, emotion* and *affect, perception, conditioning*) and measures (*eeg, startle, electrodermal*). The high centrality is likely due to a bridge function between the other clusters. More precisely, Cluster 4 (yellow) mainly included cardiovascular themes (*cardiovascular, heart rate, hypertension, blood pressure*) that were not very tightly interconnected (low density) and rather marginal—indicating the decline of research restricted to cardiovascular measures in the face of emerging ERP research. Here, Cluster 2 (green) included *ERP*, the most frequently used key term from 1984 and 2003. It reflects research on different cognitive domains (*attention, memory, detection*) across stimulus modalities (*visual, auditory*) and participant groups (*adult, child, development*) with the Mismatch Negativity (*mmn*) as one prominent example. Next to Cluster 2, Cluster 3 (blue) depicted a somewhat more specialized research field investigating the P300 ERP component (*p300*), its parameters (*latency, amplitude*), and possibly functional significance (*index, evaluation, anticipation*). Finally, Cluster 5 (purple) was small and marginal but relatively developed in comparison and might be interpreted as a theme with clinical research questions (*patient, normal*) across multiple physiological outcomes (*cardiac, respiratory, brain, pattern*).

**TABLE 5:** Key terms and density for clusters of key term co‐occurrence network—1984 to 2003.

Cluster	Key terms	Size	Centrality	Density
1	human, sleep, eeg, emotion, autonomic, startle, function, perception, affect, recording, electrodermal, facial, performance, individual differences, conditioning	15	2397 (1)	2188 (1)
2	erp, attention, visual, auditory, memory, detection, child, electrophysiological, adult, development, mmn	11	1879 (3)	1668 (4)
3	p300, stimulus, method, index, evaluation, latency, amplitude, reaction time, anticipation	9	1939 (2)	1847 (3)
4	cardiovascular, heart rate, behavior, blood pressure, stress, cognition, component, hypertension	8	1382 (5)	1580 (5)
5	scene, cardiac, pattern, patients, normal, respiratory, brain	7	1423 (4)	1857 (2)

*Note:* Key terms: key terms included in the cluster, sorted for occurrences. Size: number of key terms in the cluster. Centrality: Callon's centrality as specified by Cobo et al. (2011) with cluster rank in parentheses. Density: Callon's density as specified by Cobo et al. (2011) with cluster rank in parentheses.

Abbreviations: eeg, electroencephalography; erp, event‐related potentials; mmn, mismatch negativity.

**FIGURE 12:**
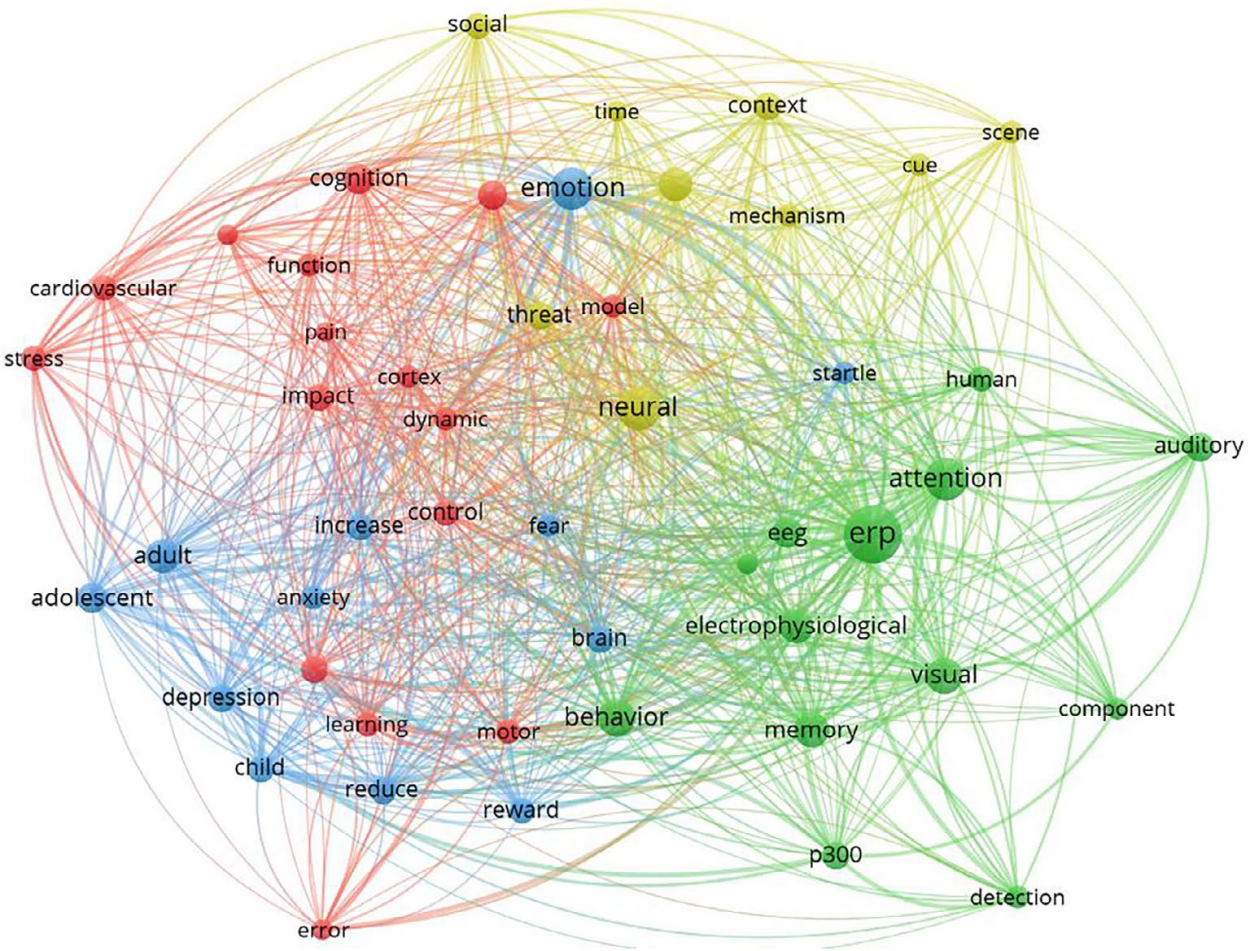
Co‐occurrence network for key terms extracted from titles of publications from 2004 to 2023.

**FIGURE 13:**
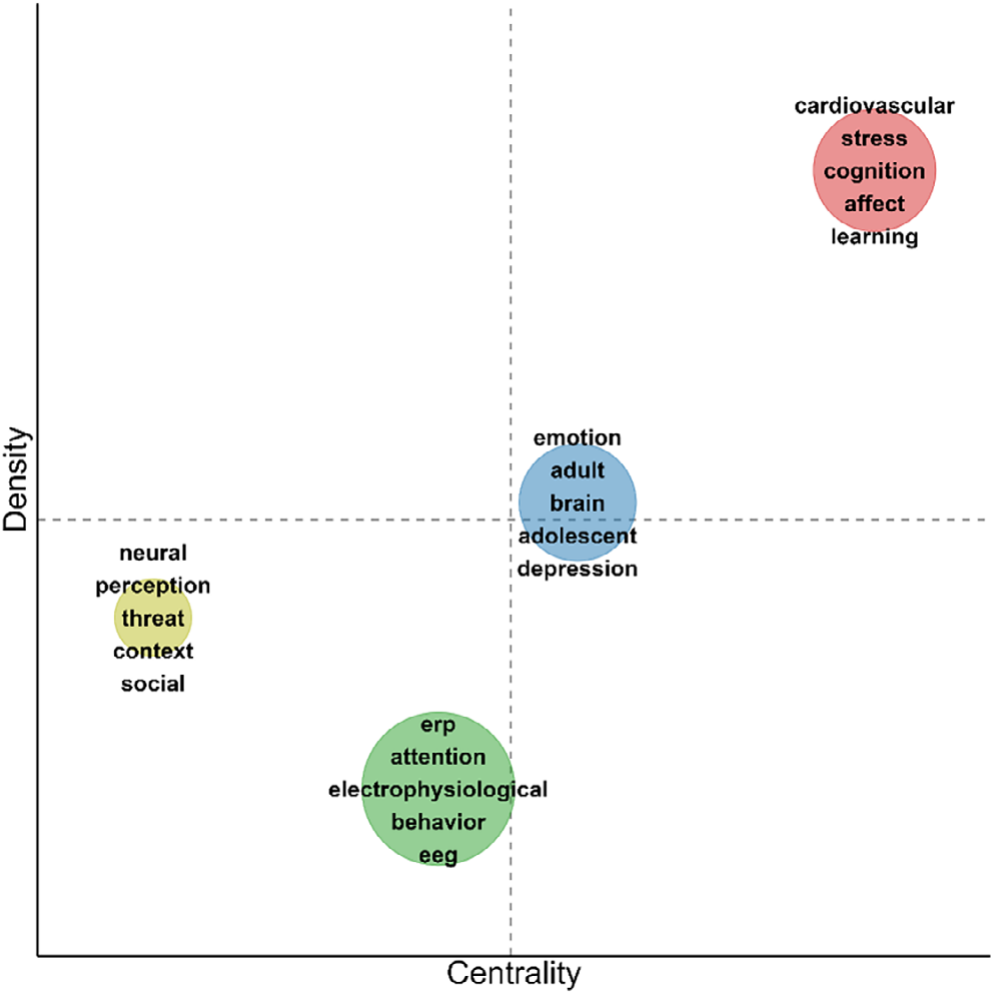
Thematic map for key term clusters from 2004 to 2023. Each cluster is depicted as a circle with its size reflecting the total occurrences across all key terms in the cluster and its position indicating Callon's centrality (*x* axis) and Callon's density (*y* axis). Dashed lines indicate unweighted average centrality and density across all clusters. For each cluster, the 5 most frequent keywords are listed.

For the time window from 2004 to 2023, 4 clusters were identified (Figure 12, Table 6). Visual inspection of the network suggested a larger intertwinement of clusters than in previous years. Notably, Cluster 1 (red) was the largest cluster with the highest values in centrality and density (Figure 13). Despite its high density, research topics were heterogeneous (*stress, cognition, affect, learning, error, pain, control, motor*), suggesting many publications aiming at integrating different fields within the cluster but also across clusters given the high centrality. Cluster 2 (green) included the most occurring key terms, *erp* and *attention*, which were closely related. Other specific cognitive processes (*memory, detection, inhibition*), were mentioned and the cluster included *behavior* in addition to electrophysiological measures (*erp, electrophysiological, eeg*). Given the cluster's content, its relatively low density, and mid‐level centrality, it might best be characterized as basic cognitive psychophysiology research. Cluster 3 (blue) can be described as research on affective‐pathological mechanisms (*emotion, depression, fear, anxiety, reward*) comparing (*increase[d], decrease[d]*) individuals and groups, investigating different age groups (*adult, adolescent, child*). The cluster had a density level somewhat above average, suggesting a developed subfield with substantial exchange. Centrality was also somewhat above average, most likely due to the term *emotion*, but also *startle, fear*, and *brain*, which provided central connections to other clusters. Finally, Cluster 4 (yellow) was the smallest and most marginal cluster. It featured key terms referring to basic stimulus processing (*perception, context, scene, cue*) in different stimulus categories (*threat, social*, potentially *social threat*). Particularly, *threat* and *neural* connected Cluster 4 to the other clusters.

**TABLE 6:** Key terms and density for clusters of key term co‐occurrence network—2004 to 2023.

Cluster	Key terms	Size	Centrality	Density
1	cardiovascular, stress, cognition, affect, learning, impact, error, performance, cardiac, cortex, function, dynamic, model, pain, control, motor	16	2466 (1)	2198 (1)
2	erp, attention, electrophysiological, behavior, eeg, visual, memory, auditory, p300, human, detection, component, inhibition	13	1993 (3)	1632 (4)
3	emotion, adult, brain, adolescent, depression, startle, increase, reduce, child, fear, anxiety, reward	12	2144 (2)	1894 (2)
4	neural, perception, threat, context, social, scene, cue, time, mechanism	9	1684 (4)	1789 (3)

*Note:* Key terms: key terms included in the cluster, sorted for occurrences. Size: number of key terms in the cluster. Centrality: Callon's centrality as specified by Cobo et al. (2011) with cluster rank in parentheses. Density: Callon's density as specified by Cobo et al. (2011) with cluster rank in parentheses.

Abbreviations: eeg, electroencephalography; erp, event‐related potentials.

## 4 Discussion

This study aimed to objectively and quantitatively analyze and outline the development and trends of *Psychophysiology*, particularly in the context of the journal's 60th anniversary. This analysis was informative given that, in addition to numerous technological and theoretical advances, *Psychophysiology* has gone through several structural changes over the past 60 years. The journal grew in terms of manuscripts published per year, going from publishing 768 to 1020 pages per volume in 2003. Also, in 2015, *Psychophysiology* ended its printed version and became an online‐only journal, which allowed the free publication of color pictures and freedom from page counts—since then, journal submissions started growing and nearly doubled. In the current work, we quantitatively assessed the journal's performance, characteristics of contributing authors, the most influential papers, and the thematic development of the journal's content over 60 years (1964–2023). The findings not only showcase the productivity and growth of psychophysiological research but also serve as a platform to provide recommendations for further advancements and improvements in the field.

The journal's performance has shown a steep increase in terms of both annual submissions and publications per year, likely reflecting growing productivity in the field and *Psychophysiology*'s adaptation to the rising research output by psychophysiological researchers worldwide (e.g., introducing Manuscript Central as the online submission system in 2006). The increasing number of submissions likely indicates enhanced productivity driven by expanding research teams and improved research infrastructure. At the same time, it is widely argued that publication pressure in academia (“publish or perish”) contributes to a rise in both submitted and published research. Such pressure may have negative consequences for research quality (Anderson et al. 2007) and the well‐being of researchers, especially early‐career researchers without tenure (Haven et al. 2019; Tijdink, Vergouwen, and Smulders 2013). Scientific journals therefore should aim for a balanced review process that adapts to increasing submission numbers without lowering quality standards. Overall, it appears that *Psychophysiology* achieves this balance rather effectively. Despite the growth in submissions at *Psychophysiology*, the increase in publication numbers has been less pronounced: while submissions have roughly quadrupled since 2000, publications have only tripled. This suggests that quality standards have been maintained, even with the higher volume of submissions. Moreover, registered reports (Keil et al. 2020) have been introduced as another tool to facilitate the publication of robust and high‐quality work. Finally, according to the current editor‐in‐chief, *Psychophysiology* remains committed to supporting and publishing programmatic work to promote rigorous research practices (Keil 2024). As one potential avenue to further increase methodological rigor, the journal may intensify the enforcement of (a) adherence of method reports to *Psychophysiology*'s guideline papers and (b) adequate, justified sample sizes to provide sufficient statistical power. Both these criteria have been reported to not be met by a substantial amount of ERP studies in psychophysiological and neuroscientific journals, including *Psychophysiology* (Clayson et al. 2019). Such a requirement of authors to submit studies with sufficiently detailed method reports and justified sample sizes would contribute to increased transparency and replicability of published research.

The upward trend of submissions and publications was accompanied by a recent rise in the journal's impact factor. After a relatively stable period from 2005 on, it has steadily increased since 2017, reflecting the growing visibility and influence of articles published in *Psychophysiology*. This trend may, on the one hand, suggest a broadening of the journal's scope (e.g., by incorporating topics such as psychopathology), thus attracting a wider audience of researchers from various fields and methodological backgrounds. On the other hand, the establishment of Open Access policies across many countries over the past 10 to 15 years has likely enabled more researchers to access a greater volume of research (Huang et al. 2020), including *Psychophysiology* publications.

Our analysis of authorship trends revealed a significant increase in the number of contributing authors per publication over the years. This trend parallels developments in other scientific fields (Larivière et al. 2015), and several factors likely contribute to this rise. One key factor is the growing prevalence of large author teams, often attributed to large‐scale collaborations. In the age of large replication projects (Klein et al. 2018; Pavlov et al. 2021), increasingly more psychophysiological studies spend larger efforts on pooling data across research groups to achieve sufficient statistical power and generalizability. For *Psychophysiology* publications, large author teams (e.g., 15 authors and more) typically publish research with clinical samples (Güntekin et al. 2022; Perez Alday et al. 2023; Radant et al. 2010; Shah et al. 2019), genetic analyses (Savage et al. 2019; Vrieze et al. 2014), longitudinal investigations (Diekfuss et al. 2020; Isler et al. 2023), or other large‐scale studies pooling data from multiple laboratories (Koenig et al. 2021). However, the rise in the median number of authors per publication suggests that large‐scale projects alone do not explain this trend. International collaborations, including bilateral partnerships, have been accounting for an increasing share of publications. Whether authors come from one research group or multiple, two key factors likely drive the growth in author teams. First, the increased complexity of research questions and the growing interdisciplinarity of research demand more specialized expertise (Hosseini et al. 2022; Larivière et al. 2015). This trend, long established in medical research—where large, interdisciplinary teams are the norm—is now becoming more prominent in psychology and psychophysiology, which have traditionally favored smaller author teams. Second, the culture of recognizing contributions may be evolving, for both positive and problematic reasons. On one hand, researchers might be added as authors without sufficient contributions (Flanagin et al. 1998; Gülen et al. 2020), possibly to bolster their track record in response to the previously mentioned publication pressures (Plummer et al. 2023). On the other hand, genuine contributions that previously may not have earned authorship—such as data collection or assistance in data analysis—are now more likely to be recognized. Guidelines that promote transparency in authorships, like the Contributor Roles Taxonomy (CRediT; Brand et al. 2015) employed by *Psychophysiology*, have raised awareness of these issues and should be further adopted by journals to clarify contributions (Hosseini et al. 2022). Regardless of the various reasons for expanding author teams, this trend highlights both the expanding opportunities and the challenges of collaboration, making it clear that working effectively in collaborative environments is a skill that should be emphasized during training (Oliver et al. 2018).

The increase in internationally co‐authored publications was particularly evident between research institutions in North America and Europe. This growth highlights the importance of international collaborations to advance innovation in psychophysiological research. Yet, despite the expansion of international partnerships, publications still predominantly come from W.E.I.R.D. countries (Western, Educated, Industrialized, Rich, and Democratic; Henrich, Heine, and Norenzayan 2010). This may be partly due to better research infrastructure (i.e., universities, research institutes, funding agencies) or higher prioritization of certain research areas by policy in these regions compared to many non‐W.E.I.R.D. countries. In addition, psychological biases among editors, peer reviewers, and readers may negatively influence the perception and impact of research from non‐W.E.I.R.D. countries (Gomez, Herman, and Parigi 2022; Harris et al. 2015). Among the top‐publishing countries in *Psychophysiology*, the United States stands out as the leading contributor, producing more than twice the number of publications compared to the second‐highest contributor, Germany. While some countries outside of North America and Europe, such as Israel, Japan, and most notably China, are among the top contributors, their relative amount of international collaboration has yet to catch up to that of Western countries. While psychophysiological research is becoming more collaborative across countries, the current results suggest that there remains an imbalance in global research contributions, with underrepresentation from non‐W.E.I.R.D. regions both in terms of overall output and collaboration. This issue was notably addressed in a special issue and paper by Lisa M. Gatzke‐Kopp, published in *Psychophysiology* (Gatzke‐Kopp 2016), which described the issue of the generalizability of findings on neural and behavioral processes across gender, racial, and socioeconomic minorities, even within the United States. Gatzke‐Kopp's work highlights the need for more inclusive research to ensure that psychophysiological findings apply to a broader and more representative population. This issue was then further discussed in a recent paper by Kissel and Friedman, which provided guidelines for reporting participants' sociodemographic characteristics in psychophysiological research (Kissel and Friedman 2023). *Psychophysiology* could promote further international diversification by encouraging (a) submissions from underrepresented countries in general and (b) international collaborations and data sharing between W.E.I.R.D. and non‐W.E.I.R.D. countries with the aim of joint publications. Such collaborations could also provide valuable training opportunities for researchers from regions with fewer research resources. In this regard, the Society for Psychophysiological Research is already offering various exchange opportunities, such as the SPR Research Training Grant, a fellowship aimed at facilitating in‐person and online mentoring by enabling applicants to visit a laboratory with expertise in a psychophysiological method not available at their home institution. Expanding such initiatives and targeting underrepresented geographical communities might contribute to strengthening global connections within the psychophysiological research community, leading to increased submissions from non‐W.E.I.R.D. countries. In parallel, achieving greater geographical diversity among contributing authors should be accompanied by increased representation of non‐W.E.I.R.D. regions on *Psychophysiology*'s editorial board. Such diversification, alongside Wiley's current policy of reducing or waiving publication fees for researchers from socioeconomically less advantaged countries, will hopefully encourage submissions from researchers in these regions. As another helpful development, AI‐assisted language editing tools provide more efficient and accessible possibilities to researchers from non‐English‐speaking countries to optimize manuscripts in case of language barriers, a solution explicitly endorsed by *Psychophysiology* (Fabiani, Keil, and Gatzke‐Kopp 2023; Keil 2024). The journal could accompany its different strategies to facilitate increased geographic diversity with systematic tracking and analyses of all authors' affiliations, as well as submission and acceptance rates based on country or geographical region. These data, in turn, could help evaluate ongoing efforts and inform future strategies to enhance equity and representation in the publication process.

On a positive note, the representation of female first/corresponding authors has seen substantial growth, increasing from less than 20% in the 1990s to over 50% in 4 of the past 11 years. This shift marks significant progress toward gender equity in psychophysiological research and reflects the increasing contributions of women to the field. However, some caution is warranted when interpreting these numbers. While the percentage of female first/corresponding authors has been close to 50% for the past 13 years, its median has only reached the 50% mark when analyzing the last four years. It remains too early to determine whether this recent trend will be sustained and, despite the progress made, there are still barriers for women in the field (Howe‐Walsh and Turnbull 2016; Kameny et al. 2013). Furthermore, the current numbers might not fully capture gender equity at more senior levels. First authorship, which is often held by corresponding authors, tends to be more common among early‐career researchers, while senior authorship positions are frequently held by more experienced, established researchers. Therefore, the increase in female first authors may not necessarily reflect equal representation among senior authors, as available data did not include information on senior authorship's gender. As more women enter and progress in their academic careers, it will be important to examine whether this trend toward increased representation extends to senior authorship, reflecting broader gender equity across higher career stages. Furthermore, research suggests that female authors tend to be cited less frequently than their male counterparts (Chatterjee and Werner 2021; Dworkin et al. 2020), which can impact the visibility and perceived impact of their work. This citation disparity could reinforce existing gender biases in academia and affect career advancement opportunities for women. To gain a better understanding of the potential challenges that women face in psychophysiological research, more detailed and nonaggregated data should be collected. This would allow for a closer examination of whether gender influences factors such as publication rates, citation counts, leadership positions, and overall recognition in the field, beyond just first authorships. Further efforts, such as initiatives to ensure equitable representation on editorial boards and peer review panels, help address these challenges and foster a more inclusive environment. Promoting transparency in the peer review process and developing policies that encourage gender equity in research leadership positions might also help advance the progress toward full gender parity in psychophysiology.

Regarding the most influential papers, we identified the fifty most locally cited works and mapped the network of connections between them. Forty‐six of the papers shared connections via citation and formed distinct clusters representing key topics that have shaped the journal, particularly in its early years. The clusters include hallmark areas of psychophysiological research such as cardiovascular psychophysiology, P300, EMG, affective picture viewing, heart rate variability, EEG guidelines, and error‐related brain activity. The earliest papers primarily focused on cardiovascular psychophysiology (Graham 1978; Obrist, Webb, and Sutterer 1969; Obrist et al. 1970, 1978), EMG (Fridlund and Cacioppo 1986; Graham 1975), and the P300 component (Duncan‐Johnson and Donchin 1977). In contrast, more recent influential works include a methodological paper on rigorous statistical analyses for ERPs (Luck and Gaspelin 2017) and a narrative review on the use of ERPs as biomarkers for depression (Proudfit 2015). These findings reflect the evolving focus of the field, from basic physiological measures to advanced methodological techniques and applications in clinical settings. Notably, many of the top‐cited publications are methodological in nature, emphasizing the importance of standardized and reliable frameworks for psychophysiological research. This includes guideline papers on the assessment and analysis of cardiovascular measures (Berntson, Cacioppo, and Quigley 1993; Berntson et al. 1997; Sherwood et al. 1990), EMG and startle (Blumenthal et al. 2005; Fridlund and Cacioppo 1986), as well as EEG/MEG (Keil et al. 2014; Picton et al. 2000; Pivik et al. 1993). Statistical methods papers have also been highly cited (Graham 1978; Junghöfer et al. 2000; Jung et al. 2000; Kiesel et al. 2008; Luck and Gaspelin 2017; Miller, Patterson, and Ulrich 1998; Vasey and Thayer 1987), highlighting the journal's role in advancing methodological rigor. This underscores the critical role that *Psychophysiology* plays in providing a platform for methodological innovation and best practices in the field. Recent guideline papers, such as those on EEG frequency and time‐frequency measures (Keil et al. 2022), pupillary measurements (Steinhauer et al. 2022), as well as heart rate and heart rate variability (Quigley et al. 2024), show the journal's continued commitment to publishing state‐of‐the‐art methodological work, with more likely to follow in the future. The structure of the citation network reveals that *Psychophysiology* fosters programmatic research. Most clusters of influential papers span over 20 to 30 years, indicating a cumulative building of knowledge. Furthermore, 46 out of the 50 most cited papers are interconnected, with almost all clusters showing links to other clusters, demonstrating effective information flow between different subfields.

Relatedly, key‐terms development highlighted a thematic evolution over time. Initially, the field focused on peripheral physiological measures, such as cardiovascular and electrodermal responses, as well as basic stimulus processing and learning like orienting, conditioning, and habituation. This trend was followed by a marked shift toward investigating more central physiological measures, including ERPs, and exploring higher‐order cognitive functions and emotional processes, even in clinical samples. Looking ahead, it will be essential to continue adopting an integrated approach that includes both neural and peripheral measures to keep on advancing our understanding of complex psychophysiological interactions and their implications for both theory and clinical practice. The importance of integrating multiple measures was also pointed out by Monica Fabiani and her 2015 Editorial, where she encouraged researchers to “re‐embody” the brain, that is, to combine multiple bodily systems to capture the complexity of human behavior and cognition (Fabiani 2015).

Co‐occurrence analyses not only reflected this thematic evolution, from peripheral physiological measures and basic stimulus processing to central neural measures and higher‐order cognitive functions, but also provided a more detailed view of the field's dynamics by mapping clusters of key terms used in article titles. The first two decades of *Psychophysiology* can be described as a foundational period for the field. Although EEG was already used, particularly in sleep research (Cluster 4) and stimulus perception studies (Cluster 5), research predominantly focused on peripheral measures, with heart rate being the most frequently used term. Cardiovascular and electrodermal measures formed distinct, specialized clusters, and while one transversal cluster (Cluster 1) connected these subfields, overlap between them was limited, allowing rather clear distinctions between research areas. From 1984 to 2003, there was a marked rise in cognitive ERP research. Although peripheral measures, especially cardiovascular ones, remained productive, they became somewhat more peripheral to the field's central concerns. During this period, ERP research, particularly focused on the P300 component, gained significant traction. Our analysis highlighted that the P300 became a dominant theme in one of the co‐occurrence clusters during this time, and it also emerged as a central topic in the citation network, with influential papers spanning from the late 1970s (Duncan‐Johnson and Donchin 1977) to the early 2000s (Kok 2001). Cognition also became a key theme in psychophysiological research, particularly in ERP studies, with *attention* and *memory* emerging as frequently used terms. The third time period, from 2004 to 2023, saw the rise of affective neuroscience and a continued integration of different research areas. *Emotion* became the most frequently used term behind *ERP*, signaling a growing interest in the affective aspects of psychophysiology. While terms like *emotion* and *cognition* were initially clustered separately, with attention and memory in one cluster and emotion in another, the boundaries between these clusters have blurred over time. The thematic overlap between research areas has increased, and today's publication landscape is more integrated than ever before. While there have always been transversal clusters acting as thematic hubs (always represented by Cluster 1), the field as a whole has continuously evolved toward a more unified and interdisciplinary network.

This study presents with some methodological limitations. First, a substantial amount of data, for example, author gender, was missing before the late 90s and between 2005 and 2007 to 2010, which limited our ability to properly trace the evolution of researchers' trends. Despite these gaps, the overall trend is clear: the role of female researchers has increased significantly over the decades, and we are approaching a more balanced ratio of submissions from male and female authors. Similarly, complete data on the countries of the authors were only available from 1998 onward. While this restricts our ability to trace the earlier internationalization of co‐authorships, the available data still provide a meaningful description of the current landscape. Second, we chose to focus on the impact factor, which has only been available since 1997. Although this restricted our analysis of the journal's performance in earlier years, the impact factor remains a standardized and widely recognized measure of a journal's relevance. As with any index, the impact factor condenses information and may not capture the full complexity of a journal's influence. To address this, we used it as one of several measures to assess *Psychophysiology*'s impact and performance in the field. Third, although we aimed for an objective assessment of the journal's thematic development, some level of subjectivity is inevitable in such analyses. Researchers must create a dictionary of key terms, deciding which terms to exclude as uninformative and which to aggregate. These decisions can depend on the specific research question. For example, it may or may not be appropriate to aggregate terms like *skin conductance level* and *skin conductance response* depending on the context. In our study, both authors independently evaluated all extracted key terms and resolved differences through discussion to minimize subjectivity. Finally, more detailed insights into thematic developments could have been achieved by analyzing author keywords or key terms from abstracts. We chose to focus on key terms extracted from article titles, as they were consistently available for all publications, whereas author keywords and abstracts were largely unavailable in Web of Science for articles before 1991 (Liu 2021). We would also argue that titles reflect the most important aspects of each study and, therefore, provide a valid indicator of the journal's content across its 60‐year history.

It is possible to speculate on what past and present publishing trends in Psychophysiology may indicate for the future. There are currently no clear signs that manuscript submissions, as reflected in submission numbers, will stop increasing, particularly if underrepresented geographical communities are better integrated into the field. While this growth may lead to a rise in low‐quality submissions, rigorous review processes could help turn this challenge into an opportunity by motivating a shift toward more “slow science” practices in order to have manuscripts accepted among the competition. Such practices prioritize programmatic, high‐quality research over output quantity as a means to publication (Frith 2020), while also fostering improved training and working conditions for early‐career researchers (Benedictus, Miedema, and Ferguson 2016). In terms of author equity, the positive trend toward increased gender equity is encouraging and may stabilize in the future. Additionally, the number of authors per paper may continue to rise as changes in how contributions are recognized and acknowledged become more widespread across generations of researchers.

Many of these publishing trends appear to intersect with the broader development of open science practices (Garrett‐Ruffin et al. 2021; Nosek et al. 2015). Although these practices were not the focus of this study, they are likely to play an important role in shaping the trends outlined here. Inclusivity and equity, such as improved gender representation and recognition of diverse contributions, inherently align with the principles of open science. Technological advances that facilitate data and material sharing may also have contributed to the growing prevalence of multi‐lab collaborations and larger author teams. Furthermore, in complex studies, these developments may create additional needs for specialized roles, such as data curators, beyond the typical responsibilities of first authors. Conversely, open science practices could also help address some of the challenges associated with these trends. Registered reports, for instance, promote methodological rigor, while preregistration, preprints, and shared data and materials enhance transparency and accountability. Openly available preprints and resources may also provide greater opportunities for researchers from underrepresented or resource‐limited regions to contribute to the field.

Taken together, this quantitative bibliometric analysis mapped the evolution and advancements that *Psychophysiology* has undergone over the past sixty years, becoming one of the leading journals for publishing innovative and rigorous research in the field. This study highlights the need to expand psychophysiological research across different geographical regions, fostering greater inclusivity across socioeconomic, cultural, and gender minorities. Broadening the diversity of research participants is essential to ensure that findings are generalizable, ultimately enriching the field and its clinical and nonclinical applications. Thematic analyses have shown a shift from a focus on peripheral to central measures, paralleled by an increasing emphasis on both cognitive and affective processes. The growing integration between previously distinct research clusters indicates a more interconnected and interdisciplinary field. This thematic convergence suggests that *Psychophysiology* should continue to prioritize interdisciplinary approaches, combining central and peripheral measures to address increasingly complex research questions in both cognitive and affective domains.

## Author Contributions

**Christian Panitz:** conceptualization, data curation, formal analysis, visualization, writing – original draft, writing – review and editing. **Carola Dell'Acqua:** conceptualization, writing – original draft, writing – review and editing.

## Conflicts of Interest

The authors declare no conflicts of interest.

## Supporting information


Data S1.

## Data Availability

The raw bibliometric data, the dictionary with synonyms and excluded key terms, as well as R data frames and scripts for statistical analyses and plotting are available on OSF: https://osf.io/6t4u5/.
