# Supplementary material for: Mapping 60 Years of Psychophysiology: A Bibliometric Analysis of Journal Performance, Authorship Trends, and Thematic Evolution
Source: Psychophysiology. 2025 Feb 2;62(2):e70002. doi: 10.1111/psyp.70002 (PMC11788248; doi:10.1111/psyp.70002)
Supplement: Supplementary file 1 — Data S1. [file PSYP-62-e70002-s001.docx]

Supplement

# Mapping 60 years of Psychophysiology: A bibliometric analysis of journal performance, authorship trends, and thematic evolution

**Author Team Size**

**
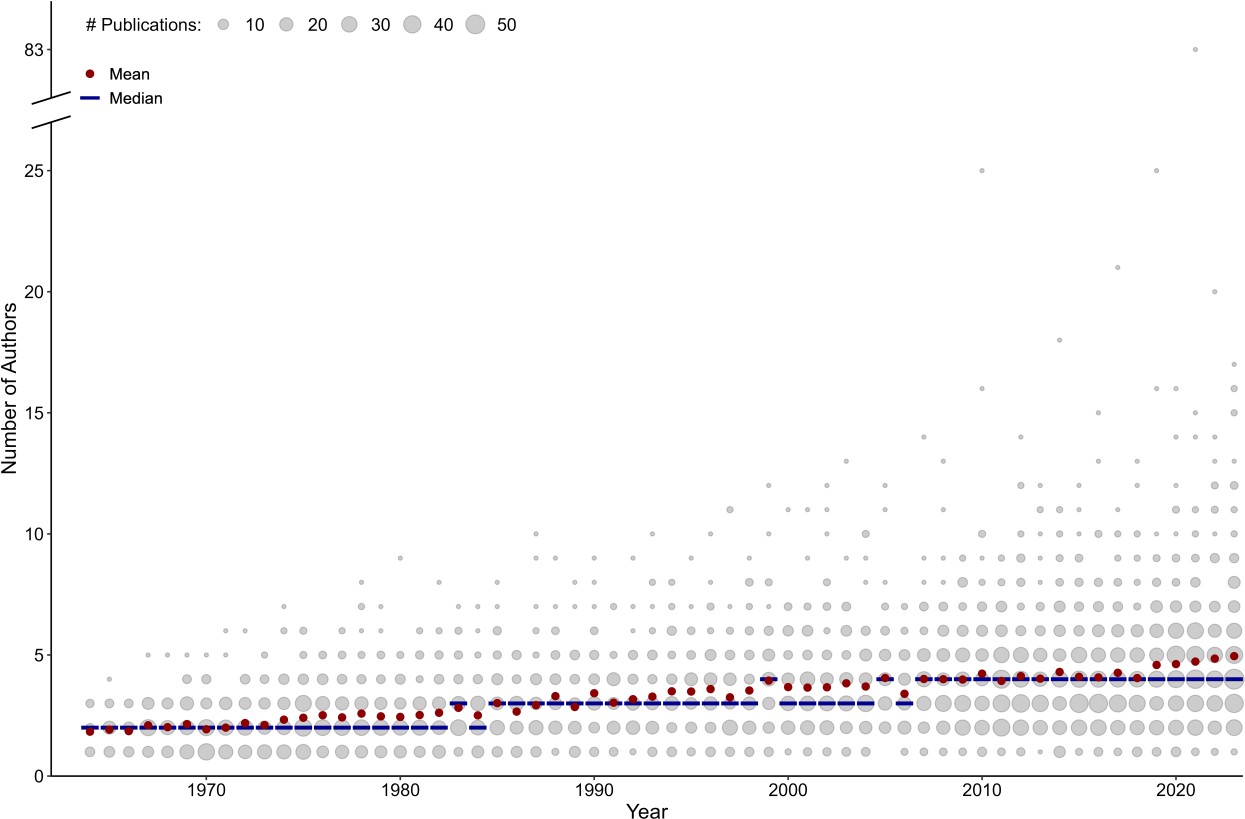
**

**Supplementary Figure 1**. Mean (red dots) and median (blue bars) number of authors per publication by publication year.

**Supplementary Table 1.** Mean and median author team sizes from 1964 to 2023.

| **Year** | **Mean** | **Median** | **Year** | **Mean** | **Median** |
| --- | --- | --- | --- | --- | --- |
| 1964 | 1.83 | 2 | 1994 | 3.50 | 3 |
| 1965 | 1.92 | 2 | 1995 | 3.49 | 3 |
| 1966 | 1.86 | 2 | 1996 | 3.59 | 3 |
| 1967 | 2.10 | 2 | 1997 | 3.25 | 3 |
| 1968 | 2.02 | 2 | 1998 | 3.53 | 3 |
| 1969 | 2.15 | 2 | 1999 | 3.94 | 4 |
| 1970 | 1.93 | 2 | 2000 | 3.67 | 3 |
| 1971 | 2.00 | 2 | 2001 | 3.65 | 3 |
| 1972 | 2.19 | 2 | 2002 | 3.67 | 3 |
| 1973 | 2.11 | 2 | 2003 | 3.83 | 3 |
| 1974 | 2.33 | 2 | 2004 | 3.70 | 3 |
| 1975 | 2.41 | 2 | 2005 | 4.05 | 4 |
| 1976 | 2.51 | 2 | 2006 | 3.40 | 3 |
| 1977 | 2.42 | 2 | 2007 | 4.01 | 4 |
| 1978 | 2.58 | 2 | 2008 | 4.00 | 4 |
| 1979 | 2.47 | 2 | 2009 | 3.99 | 4 |
| 1980 | 2.44 | 2 | 2010 | 4.23 | 4 |
| 1981 | 2.52 | 2 | 2011 | 3.93 | 4 |
| 1982 | 2.61 | 2 | 2012 | 4.13 | 4 |
| 1983 | 2.82 | 3 | 2013 | 4.02 | 4 |
| 1984 | 2.51 | 2 | 2014 | 4.30 | 4 |
| 1985 | 3.01 | 3 | 2015 | 4.10 | 4 |
| 1986 | 2.66 | 3 | 2016 | 4.07 | 4 |
| 1987 | 2.93 | 3 | 2017 | 4.26 | 4 |
| 1988 | 3.30 | 3 | 2018 | 4.05 | 4 |
| 1989 | 2.85 | 3 | 2019 | 4.59 | 4 |
| 1990 | 3.42 | 3 | 2020 | 4.62 | 4 |
| 1991 | 3.03 | 3 | 2021 | 4.97 | 4 |
| 1992 | 3.17 | 3 | 2022 | 4.85 | 4 |
| 1993 | 3.28 | 3 | 2023 | 4.95 | 4 |

# Publications by Countries

**Supplementary Table 2***.* Accumulated publication numbers for countries of authors’ research institutions from 1998 to 2023*.*

| **Country** | **Publications** | **Country** | **Publications** | **Country** | **Publications** |
| --- | --- | --- | --- | --- | --- |
| USA | 3,710 | Brazil | 63 | Argentina | 6 |
| Germany | 1,273 | Sweden | 60 | Luxembourg | 6 |
| China | 785 | Ireland | 50 | Romania | 6 |
| UK | 591 | Poland | 50 | Colombia | 4 |
| Netherlands | 490 | New Zealand | 48 | Estonia | 4 |
| Canada | 437 | Portugal | 26 | Malaysia | 4 |
| Australia | 406 | Russia | 26 | Slovenia | 4 |
| Spain | 283 | Korea | 25 | Bulgaria | 3 |
| France | 244 | Denmark | 22 | Iran | 3 |
| Italy | 238 | Türkiye | 22 | Ukraine | 3 |
| Switzerland | 208 | Chile | 21 | Cuba | 2 |
| Belgium | 153 | South Africa | 17 | India | 2 |
| Japan | 151 | Czech Republic | 15 | Saudi Arabia | 2 |
| Finland | 146 | Singapore | 10 | United Arab Emirates | 2 |
| Israel | 123 | Cyprus | 9 | Egypt | 1 |
| Hungary | 102 | Greece | 7 | Iceland | 1 |
| Austria | 100 | Mexico | 7 | Jordan | 1 |
| Norway | 67 | Uruguay | 7 | Qatar | 1 |

# International Collaborations

**Supplementary Table 3.** Number of publications with co-authors from different countries.

| Country | USA | Germany | China | UK | Nether- lands | Canada | Australia | Spain | France | Italy | Switzer- land | Belgium | Japan | Finland | Israel | Total |
| --- | --- | --- | --- | --- | --- | --- | --- | --- | --- | --- | --- | --- | --- | --- | --- | --- |
| USA | . | 115 | 56 | 60 | 57 | 64 | 29 | 35 | 21 | 22 | 31 | 9 | 8 | 10 | 18 | 535 |
| Germany | 115 | . | 21 | 70 | 42 | 17 | 17 | 16 | 14 | 17 | 37 | 17 | 6 | 6 | 7 | 402 |
| China | 56 | 21 | . | 16 | 11 | 8 | 7 | 1 | 1 | 2 | 4 | 2 | 3 | 7 | 1 | 140 |
| UK | 60 | 70 | 16 | . | 29 | 20 | 24 | 7 | 5 | 17 | 21 | 10 | 1 | 4 | 3 | 287 |
| Netherlands | 57 | 42 | 11 | 29 | . | 8 | 11 | 4 | 5 | 8 | 5 | 24 | 2 | . | 5 | 211 |
| Canada | 64 | 17 | 8 | 20 | 8 | . | 7 | 2 | 8 | 17 | 3 | 3 | 2 | 2 | 4 | 165 |
| Australia | 29 | 17 | 7 | 24 | 11 | 7 | . | 7 | 5 | 2 | 5 | 2 | 1 | 1 | . | 118 |
| Spain | 35 | 16 | 1 | 7 | 4 | 2 | 7 | . | 2 | 8 | 2 | 2 | 1 | 2 | 2 | 91 |
| France | 21 | 14 | 1 | 5 | 5 | 8 | 5 | 2 | . | 7 | 5 | 6 | 2 | 2 | 2 | 85 |
| Italy | 22 | 17 | 2 | 17 | 8 | 17 | 2 | 8 | 7 | . | 3 | 6 | 1 | 1 | 2 | 113 |
| Switzerland | 31 | 37 | 4 | 21 | 5 | 3 | 5 | 2 | 5 | 3 | . | 3 | 1 | 1 | 2 | 123 |
| Belgium | 9 | 17 | 2 | 10 | 24 | 3 | 2 | 2 | 6 | 6 | 3 | . | 1 | . | 1 | 86 |
| Japan | 8 | 6 | 3 | 1 | 2 | 2 | 1 | 1 | 2 | 1 | 1 | 1 | . | 2 | 1 | 32 |
| Finland | 10 | 6 | 7 | 4 | . | 2 | 1 | 2 | 2 | 1 | 1 | . | 2 | . | . | 38 |
| Israel | 18 | 7 | 1 | 3 | 5 | 4 | . | 2 | 2 | 2 | 2 | 1 | 1 | . | . | 48 |

*Note: The total number of publications by country is the sum of all publications with other countries in this table (Top 15 publishing countries), collaborations with other countries do not count towards this number.*

# Key Term Trends Over Years

**
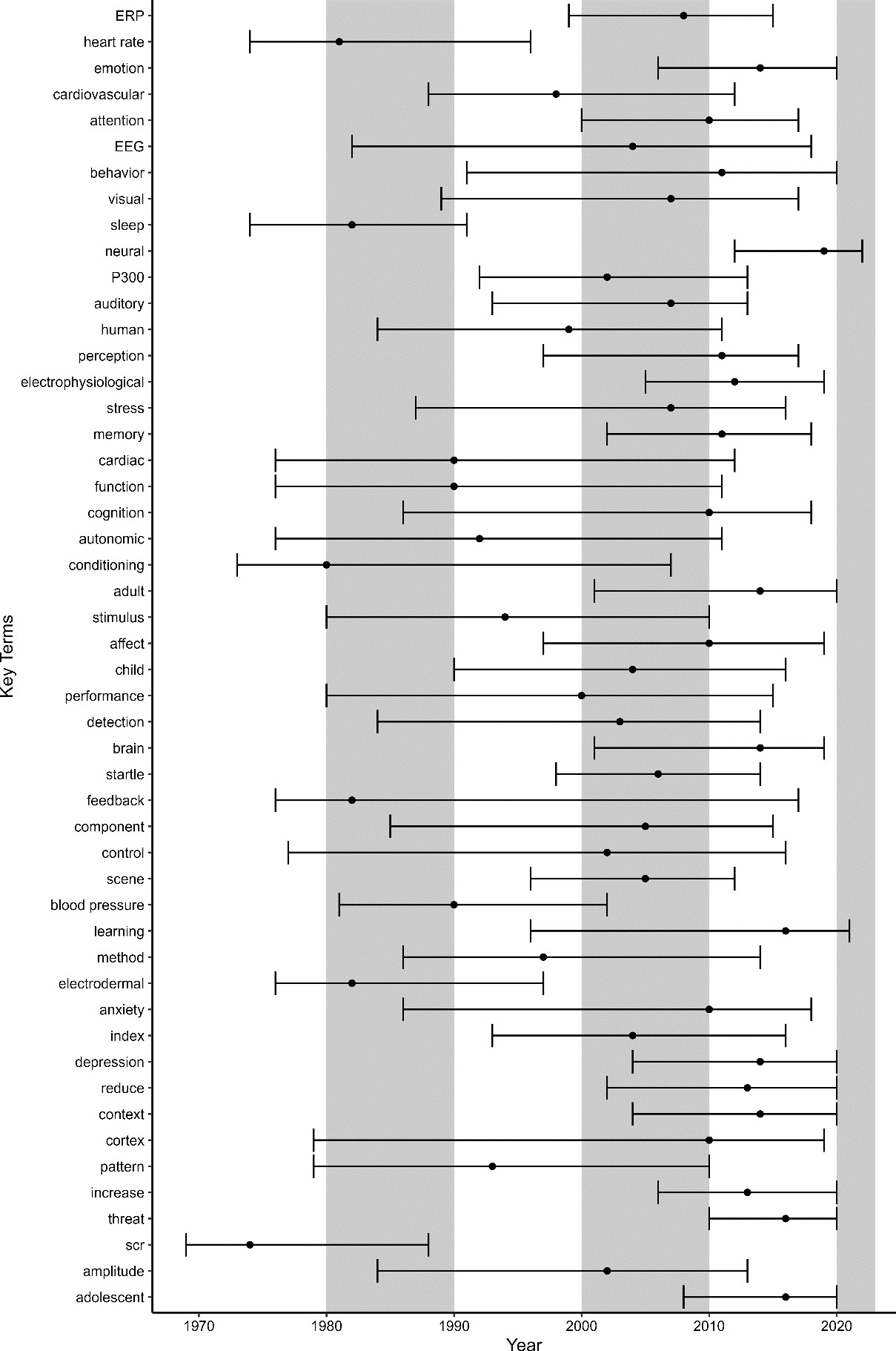
**

**Supplementary Figure 2.** The 50 most frequent key terms of all time (1964 to 2023) in descending order of total occurrences. Dots indicate the median publication year of all articles using the key term, whiskers reflect the 25th and 75th percentiles.

# Historical Direct Citations Network

**Supplementary Table 4.** Clusters of publications in the Historical Direct Citation Network

| **Cluster** | **Year** | **First Author** | **Title** | **Local Citations** | **Global Citations** | **DOI** |
| --- | --- | --- | --- | --- | --- | --- |
| 1 | 1969 | Obrist PA | Heart Rate and Somatic Changes During Aversive Conditioning and a Simple Reaction Time Task | 45 | 165 | 10.1111/j.1469-  8986.1969.tb02872.x |
| 1 | 1970 | Obrist PA | Cardiac-Somatic Relationship - Some Reformulations | 50 | 268 | 10.1111/j.1469-  8986.1970.tb02246.x |
| 1 | 1975 | Graham FK | More or Less Startling Effects of Weak Pre-Stimulation | 78 | 1,003 | 10.1111/j.1469-  8986.1975.tb01284.x |
| 1 | 1978 | Obrist PA | Relationship Among Heart Rate, Carotid dP/dt, and Blood Pressure in Humans as a Function of Type of Stress | 64 | 309 | 10.1111/j.1469-  8986.1978.tb01344.x |
| 1 | 1986 | Sherwood A | Evaluation of Beta-Adrenergic Influences on Cardiovascular and Metabolic Adjustments to Physical and Psychological Stress | 68 | 265 | 10.1111/j.1469-  8986.1986.tb00602.x |
| 1 | 1990 | Kelsey RM | An Evaluation of the Ensemble Averaged Impedance Cardiogram | 50 | 168 | 10.1111/j.1469-  8986.1990.tb02173.x |
| 1 | 1990 | Sherwood A | Methodological Guidelines for Impedance Cardiography | 132 | 978 | 10.1111/j.1469-  8986.1990.tb02171.x |
| 2 | 1977 | Duncan-Johnson CC | Quantifying Surprise - Variation of Event-Related Potentials with Subjective Probability | 112 | 935 | 10.1111/j.1469-  8986.1977.tb01312.x |
| 2 | 1984 | Magliero A | On the Dependence of P300 Latency on Stimulus Evaluation Processes | 50 | 509 | 10.1111/j.1469-  8986.1984.tb00201.x |
| 2 | 1986 | Johnson R | For Distinguished Early Career Contribution to Psychophysiology - Award Address, 1985 - a Triarchic Model of P300 Amplitude | 107 | 848 | 10.1111/j.1469-  8986.1986.tb00649.x |

| **Cluster** | **Year** | **First Author** | **Title** | **Local Citations** | **Global Citations** | **DOI** |
| --- | --- | --- | --- | --- | --- | --- |
| 2 | 1997 | Verleger R | On the Utility of P3 Latency as an Index of Mental Chronometry | 51 | 431 | 10.1111/j.1469-  8986.1997.tb02125.x |
| 2 | 2001 | Kok A | On the Utility of P3 Amplitude as a Measure of Processing Capacity | 77 | 1,282 | 10.1017/S0048577201990  559 |
| 2 | 2007 | Hajcak G | It's Worse Than You Thought: The Feedback Negativity and Violations of Reward Prediction in Gambling Tasks | 65 | 431 | 10.1111/j.1469-  8986.2007.00567.x |
| 2 | 2008 | Kiesel A | Measurement of ERP Latency Differences: A Comparison of Single-Participant and Jackknife-Based Scoring Methods | 49 | 321 | 10.1111/j.1469-  8986.2007.00618.x |
| 3 | 1986 | Fridlund AJ | Guidelines for Human Electromyographic Research | 89 | 1,323 | 10.1111/j.1469-  8986.1986.tb00676.x |
| 3 | 1990 | Bradley MM | Startle Reflex Modification - Emotion or Attention | 58 | 328 | 10.1111/j.1469-  8986.1990.tb01966.x |
| 3 | 1993 | Lang PJ | Looking at Pictures - Affective, Facial, Visceral, and Behavioral Reactions | 109 | 2,308 | 10.1111/j.1469-  8986.1993.tb03352.x |
| 3 | 1996 | Cuthbert BN | Probing Picture Perception: Activation and Emotion | 49 | 330 | 10.1111/j.1469-  8986.1996.tb02114.x |
| 3 | 2005 | Blumenthal TD | Committee Report: Guidelines for Human Startle Eyeblink Electromyographic Studies | 95 | 905 | 10.1111/j.1469-  8986.2005.00271.x |
| 4 | 1978 | Graham FK | Constraints on Measuring Heart Rate and Period Sequentially Through Real and Cardiac Time | 52 | 180 | 10.1111/j.1469-  8986.1978.tb01422.x |
| 4 | 1991 | Grossman P | Prediction of Tonic Parasympathetic Cardiac Control Using Respiratory Sinus Arrhythmia - the Need for Respiratory Control | 59 | 391 | 10.1111/j.1469-  8986.1991.tb00412.x |
| 4 | 1993 | Berntson GG | Respiratory Sinus Arrhythmia - Autonomic Origins, Physiological Mechanisms, and Psychophysiological Implications | 60 | 695 | 10.1111/j.1469-  8986.1993.tb01731.x |

| **Cluster** | **Year** | **First Author** | **Title** | **Local Citations** | **Global Citations** | **DOI** |
| --- | --- | --- | --- | --- | --- | --- |
| 4 | 1997 | Berntson GG | Heart Rate Variability: Origins, Methods, and Interpretive Caveats | 130 | 2,788 | 10.1111/j.1469-  8986.1997.tb02140.x |
| 5 | 1991 | Woldorff MG | The Effects of Channel-Selective Attention on the Mismatch Negativity Wave Elicited by Deviant Tones | 46 | 307 | 10.1111/j.1469-  8986.1991.tb03384.x |
| 5 | 1994 | Luck SJ | Electrophysiological Correlates of Feature Analysis During Visual Search | 107 | 1,090 | 10.1111/j.1469-  8986.1994.tb02218.x |
| 5 | 2008 | Folstein JR | Influence of Cognitive Control and Mismatch on the N2 Component of the ERP: A Review | 111 | 1,875 | 10.1111/j.1469-  8986.2007.00602.x |
| 5 | 2008 | Holroyd CB | The Feedback Correct-Related Positivity: Sensitivity of the Event-Related Brain Potential to Unexpected Positive Feedback | 67 | 474 | 10.1111/j.1469-  8986.2008.00668.x |
| 5 | 2015 | Proudfit GH | The Reward Positivity: From Basic Research on Reward to a Biomarker for Depression | 76 | 606 | 10.1111/psyp.12370 |
| 6 | 1987 | Vasey MW | The Continuing Problem of False Positives in Repeated Measures ANOVA in Psychophysiology - a Multivariate Solution | 130 | 988 | 10.1111/j.1469-  8986.1987.tb00324.x |
| 6 | 1989 | Coles MGH | Modern Mind-Brain Reading - Psychophysiology, Physiology, and Cognition | 76 | 725 | 10.1111/j.1469-  8986.1989.tb01916.x |
| 6 | 1993 | Pivik RT | Guidelines for the Recording and Quantitative Analysis of Electroencephalographic Activity in Research Contexts | 50 | 545 | 10.1111/j.1469-  8986.1993.tb02081.x |
| 6 | 1998 | Miller J | Jackknife-Based Method for Measuring LRP Onset Latency Differences | 71 | 513 | 10.1111/1469-  8986.3510099 |
| 6 | 2000 | Picton TW | Guidelines for Using Human Event-Related Potentials to Study Cognition: Recording Standards and Publication Criteria | 72 | 1,587 | 10.1017/S0048577200000  305 |
| 6 | 2000 | Jung TP | Removing Electroencephalographic Artifacts by Blind Source Separation | 60 | 2,471 | 10.1017/S0048577200980  259 |

| **Cluster** | **Year** | **First Author** | **Title** | **Local Citations** | **Global Citations** | **DOI** |
| --- | --- | --- | --- | --- | --- | --- |
| 6 | 2001 | Ulrich R | Using the Jackknife-Based Scoring Method for Measuring LRP Onset Effects in Factorial Designs | 49 | 355 | 10.1017/S0048577201000  610 |
| 6 | 2014 | Keil A | Committee Report: Publication Guidelines and Recommendations for Studies Using Electroencephalography and Magnetoencephalography | 51 | 455 | 10.1111/psyp.12147 |
| 6 | 2017 | Luck SJ | How to Get Statistically Significant Effects in Any ERP Experiment (and Why You Shouldn't) | 58 | 732 | 10.1111/psyp.12639 |
| 7 | 1987 | Näätänen R | The N1 Wave of the Human Electric and Magnetic Response to Sound - a Review and an Analysis of the Component Structure | 152 | 2,600 | 10.1111/j.1469-  8986.1987.tb00311.x |
| 7 | 2000 | Vogel EK | The Visual N1 Component as an Index of a Discrimination Process | 45 | 805 | 10.1111/1469-  8986.3720190 |
| 7 | 2000 | Junghöfer M | Statistical Control of Artifacts in Dense Array EEG/MEG Studies | 46 | 458 | 10.1111/1469-  8986.3740523 |
| 7 | 2000 | Schupp HT | Affective Picture Processing: The Late Positive Potential Is Modulated by Motivational Relevance | 61 | 1,054 | 10.1111/1469-  8986.3720257 |
| 7 | 2002 | Keil A | Large-Scale Neural Correlates of Affective Picture Processing | 46 | 533 | 10.1017/S0048577202394  162 |
| 7 | 2009 | Bradley MM | Natural Selective Attention: Orienting and Emotion | 53 | 675 | 10.1111/j.1469-  8986.2008.00702.x |
| 8 | 2001 | Nieuwenhuis S | Error-Related Brain Potentials Are Differentially Related to Awareness of Response Errors: Evidence from an Antisaccade Task | 52 | 898 | 10.1111/1469-  8986.3850752 |
| 8 | 2005 | Hajcak G | On the ERN and the Significance of Errors | 53 | 452 | 10.1111/j.1469-  8986.2005.00270.x |
| 8 | 2009 | Olvet DM | The Stability of Error-Related Brain Activity with Increasing Trials | 49 | 370 | 10.1111/j.1469-  8986.2009.00848.x |

*Note. Year = Publication year. Local Citations = Times cited by other publications from the current dataset (i.e. Psychophysiology publications from 1964 to 2023). Global citations = Times cited by any other publication. Data was downloaded on September 6, 2024.*

# Key Term Co-Occurrences (1964-1973)

**
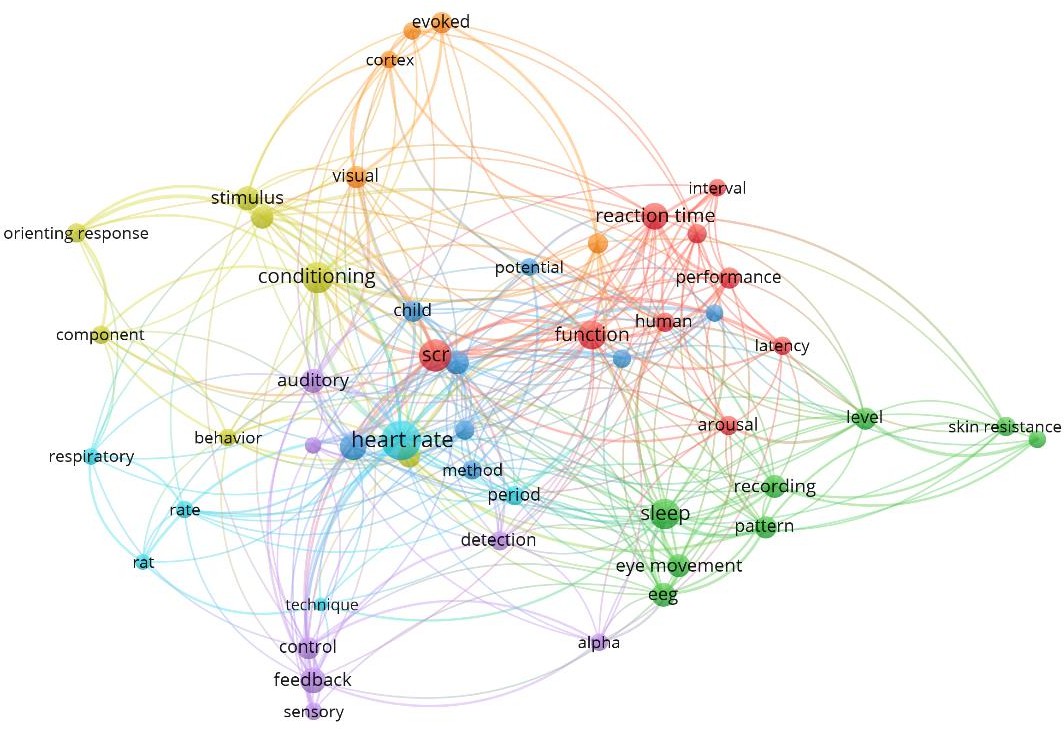
**

**Supplementary Figure 3.** Co-occurrence network for key terms extracted from publication titles (1964-1973).

**Supplementary Table 5.** Key terms and density for clusters of key term co-occurrence network (1964-1973).

| **Cluster** | **Key Terms** | **Size** | **Centrality** | **Density** |
| --- | --- | --- | --- | --- |
| 1 | scr, reaction time, human, function, performance, arousal, interval, latency, normal | 9 | 1671 (1) | 1685 (7) |
| 2 | sleep, eeg, eye movement, level, skin resistance, recording, pattern, skin potential | 8 | 1053 (5) | 1769 (6) |
| 3 | cardiac, electrodermal, differential, cognition, child, skin conductance, method, potential | 8 | 1335 (2) | 1903 (2) |
| 4 | conditioning, autonomic, orienting response, habituation, stimulus, behavior, component | 7 | 1021 (6) | 1770 (5) |
| 5 | feedback, auditory, control, detection, alpha, sensory, spontaneous | 7 | 1204 (4) | 1820 (4) |
| 6 | heart rate, period, rat, rate, technique, respiratory | 6 | 1232 (3) | 1882 (3) |
| 7 | evoked, signal, visual, cortex, average | 5 | 794 (7) | 2196 (1) |

***Note****. Key terms: key terms included in the cluster, sorted for occurrences. Size: number of key terms in the cluster. Centrality: Callon’s centrality as specified by Cobo et al. (2011) with cluster rank in parentheses. Density: Callon’s density as specified by Cobo et al. (2011) with cluster rank in parentheses.*

*
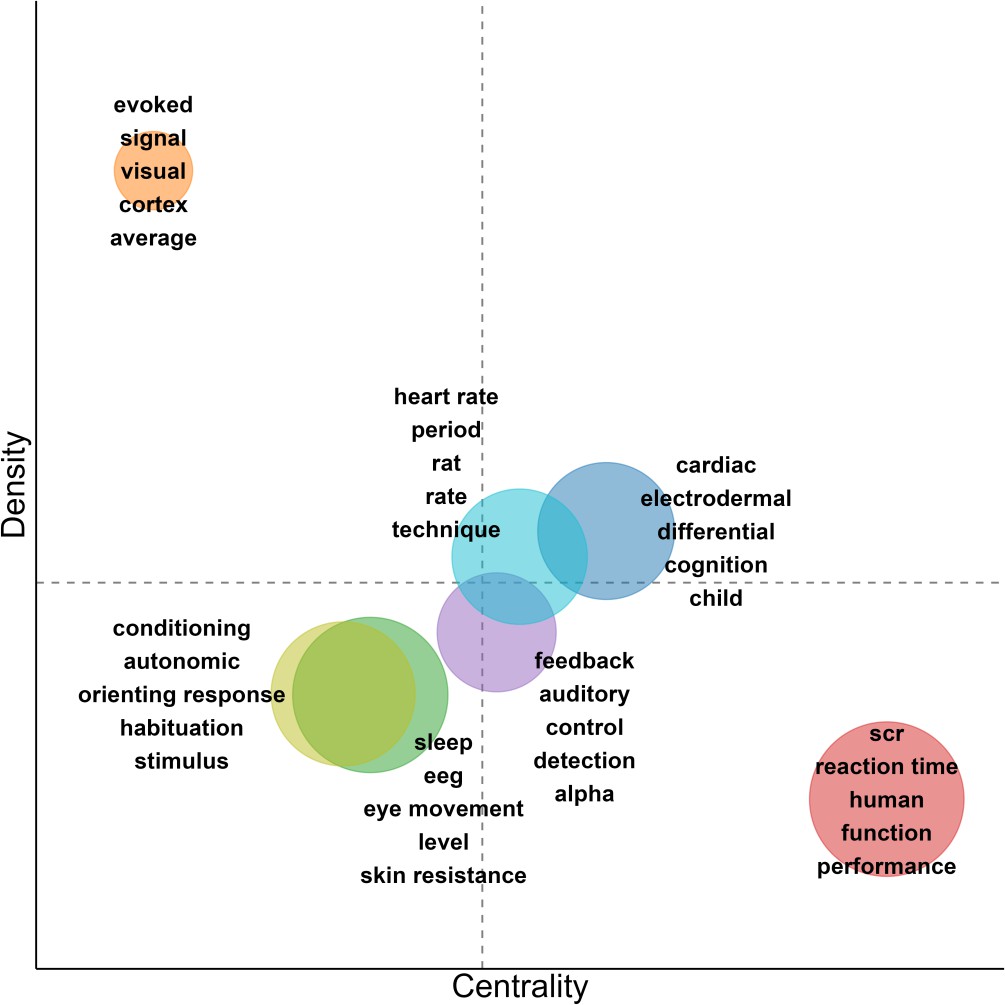
*

**Supplementary Figure 4.** Thematic map for key term clusters from 1964 to 1973. Each cluster is depicted as a circle with its size reflecting the total occurrences across all key terms in the cluster and its position indicating Callon’s centrality (x axis) and Callon’s density (y axis). Dashed lines indicate unweighted average centrality and density across all clusters. For each cluster, the 5 most frequent keywords are listed.

# Key Term Co-Occurrences (1974-1983)

**
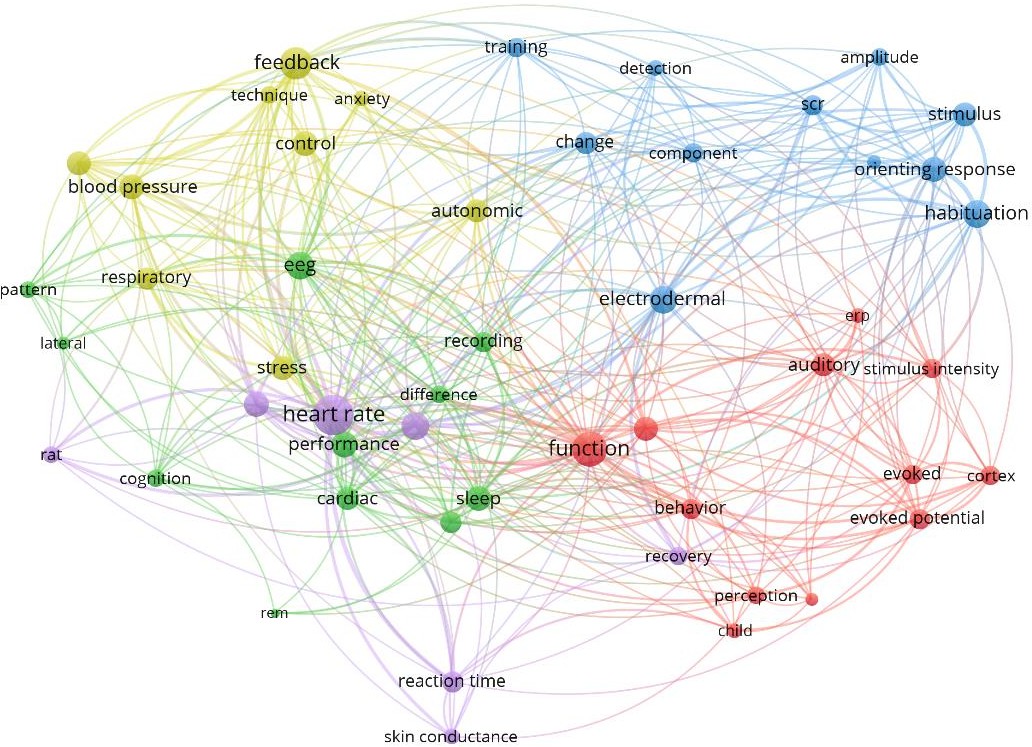
**

**Supplementary Figure 5.** Co-occurrence network for key terms extracted from publication titles (1974-1983).

**Supplementary Table 6.** Key terms and density for clusters of key term co-occurrence network (1974-1983).

| **Cluster** | **Key Terms** | **Size** | **Centrality** | **Density** |
| --- | --- | --- | --- | --- |
| 1 | function, evoked potential, visual, auditory, erp, evoked, perception, behavior, eda, child, cortex, stimulus intensity | 12 | 2452 (1) | 1944 (2) |
| 2 | sleep, eeg, cardiac, performance, recording, human, rem, pattern, cognition, difference, lateral | 11 | 1968 (2) | 1878 (3) |
| 3 | electrodermal, habituation, stimulus, orienting response, scr, detection, change, component, p300, training, amplitude | 11 | 1804 (3) | 2140 (1) |
| 4 | feedback, autonomic, blood pressure, biofeedback, control, stress, respiratory, anxiety, technique | 9 | 1616 (4) | 1549 (4) |
| 5 | heart rate, conditioning, cardiovascular, reaction time, skin conductance, rat, recovery | 7 | 1485 (5) | 1462 (5) |

***Note****. Key terms: key terms included in the cluster, sorted for occurrences. Size: number of key terms in the cluster. Centrality: Callon’s centrality as specified by Cobo et al. (2011) with cluster rank in parentheses. Density: Callon’s density as specified by Cobo et al. (2011) with cluster rank in parentheses.*


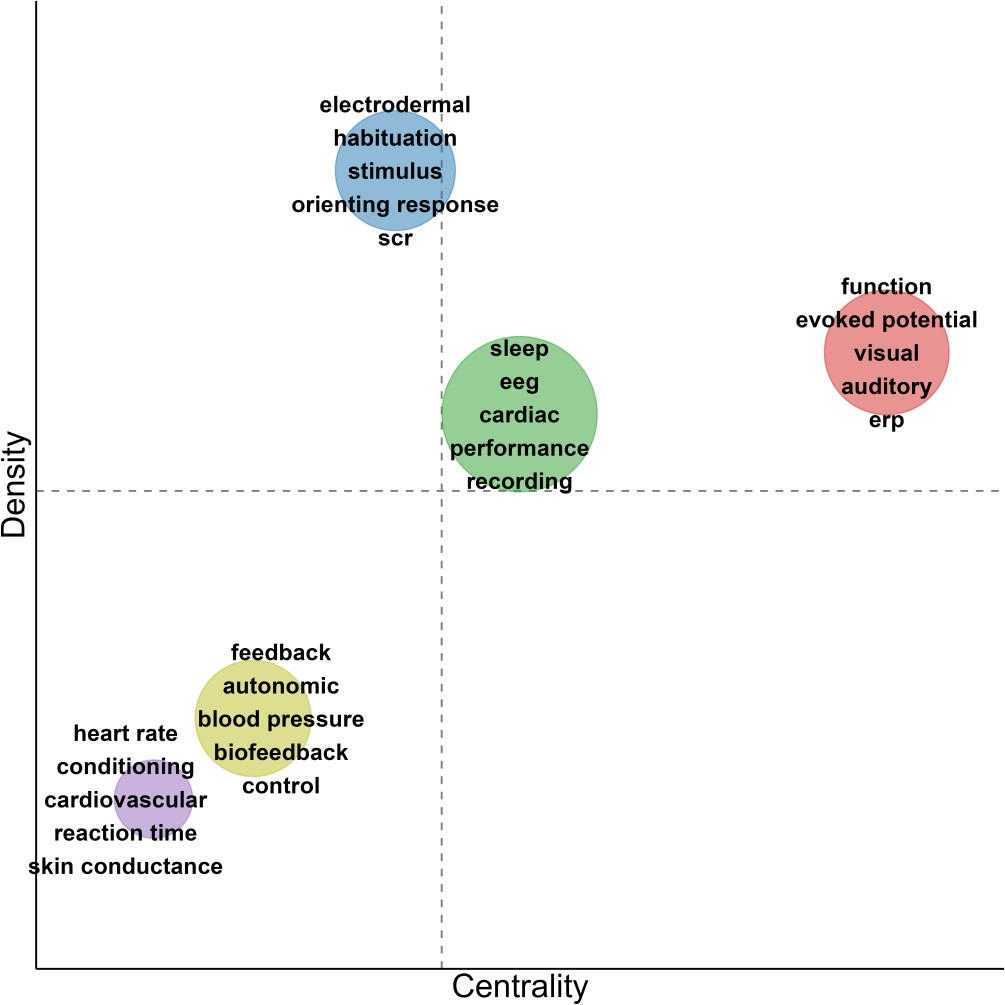


**Supplementary Figure 6.** Thematic map for key term clusters from 1974 to 1983. Each cluster is depicted as a circle with its size reflecting the total occurrences across all key terms in the cluster and its position indicating Callon’s centrality (x axis) and Callon’s density (y axis). Dashed lines indicate unweighted average centrality and density across all clusters. For each cluster, the 5 most frequent keywords are listed.

# Key Term Co-Occurrences (1984-1993)

**
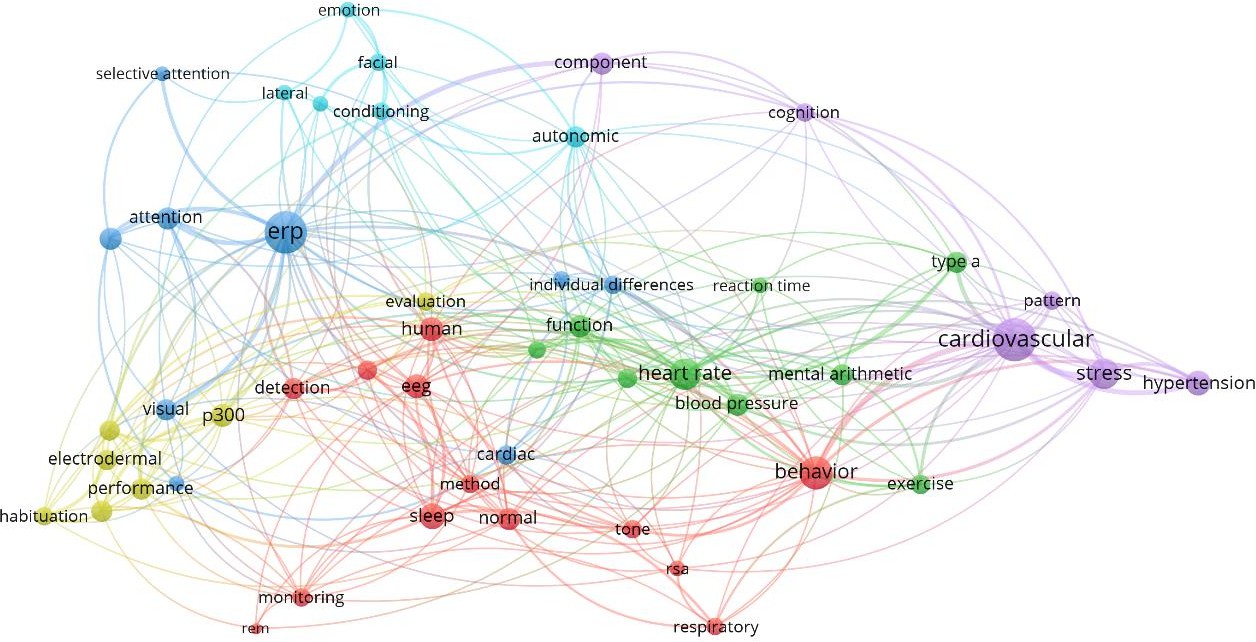
**

**Supplementary Figure 7.** Co-occurrence network for key terms extracted from publication titles (1984-1993).

**Supplementary Table 7.** Key terms and density for clusters of key term co-occurrence network (1984-1993).

| **Cluster** | **Key Terms** | **Size** | **Centrality** | **Density** |
| --- | --- | --- | --- | --- |
| 1 | sleep, behavior, human, detection, eeg, method, recording, normal, monitoring, rem, respiratory, rsa, tone | 13 | 2198 (1) | 2010 (3) |
| 2 | heart rate, blood pressure, function, exercise, type a, anxiety, mental arithmetic, reaction time, change | 9 | 1621 (3) | 1865 (4) |
| 3 | erp, attention, cardiac, visual, auditory, individual differences, patients, perception, selective attention | 9 | 1719 (2) | 1705 (5) |
| 4 | p300, stimulus, electrodermal, performance, latency, habituation, evaluation | 7 | 1091 (4) | 2074 (2) |
| 5 | cardiovascular, stress, component, cognition, hypertension, pattern | 6 | 659 (6) | 1463 (6) |
| 6 | autonomic, facial, emotion, conditioning, emg, lateral | 6 | 889 (5) | 2782 (1) |

***Note****. Key terms: key terms included in the cluster, sorted for occurrences. Size: number of key terms in the cluster. Centrality: Callon’s centrality as specified by Cobo et al. (2011) with cluster rank in parentheses. Density: Callon’s density as specified by Cobo et al. (2011) with cluster rank in parentheses.*


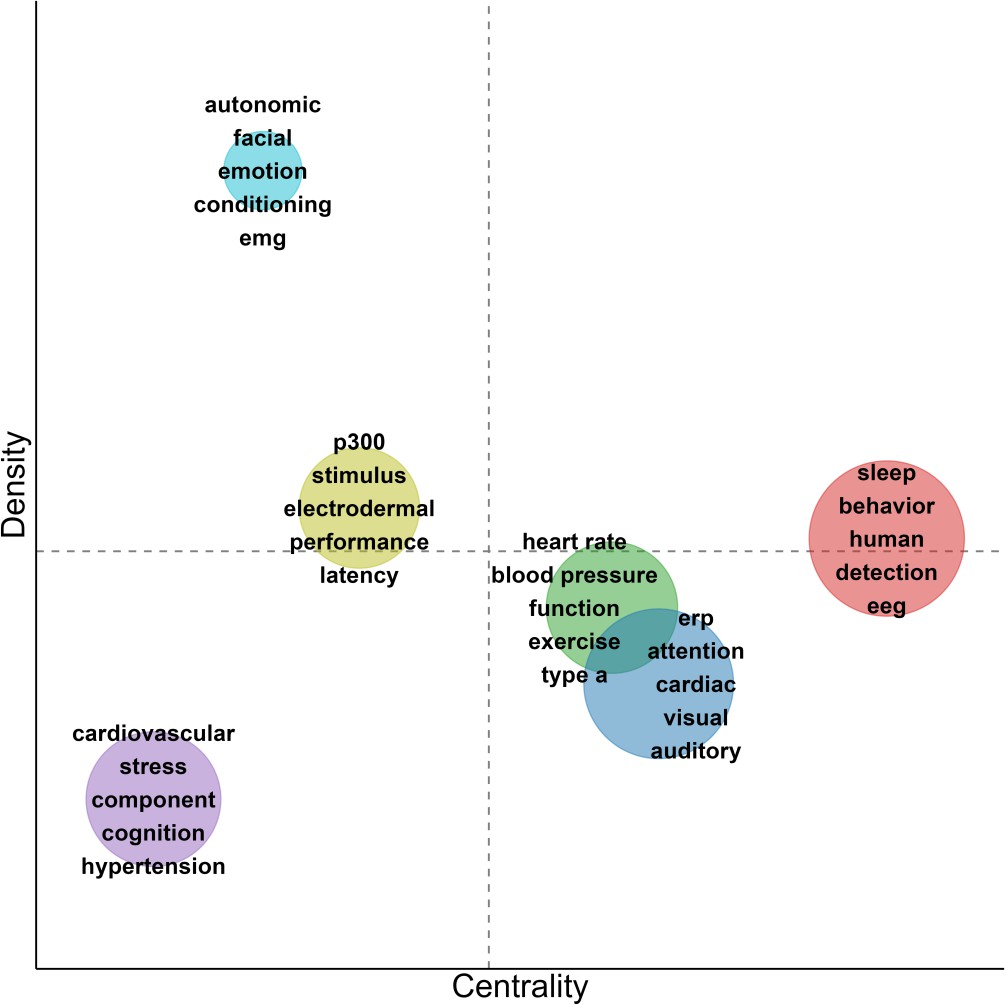


**Supplementary Figure 8.** Thematic map for key term clusters from 1984 to 1993. Each cluster is depicted as a circle with its size reflecting the total occurrences across all key terms in the cluster and its position indicating Callon’s centrality (x axis) and Callon’s density (y axis). Dashed lines indicate unweighted average centrality and density across all clusters. For each cluster, the 5 most frequent keywords are listed.

# Key Term Co-Occurrences (1994-2003)

**
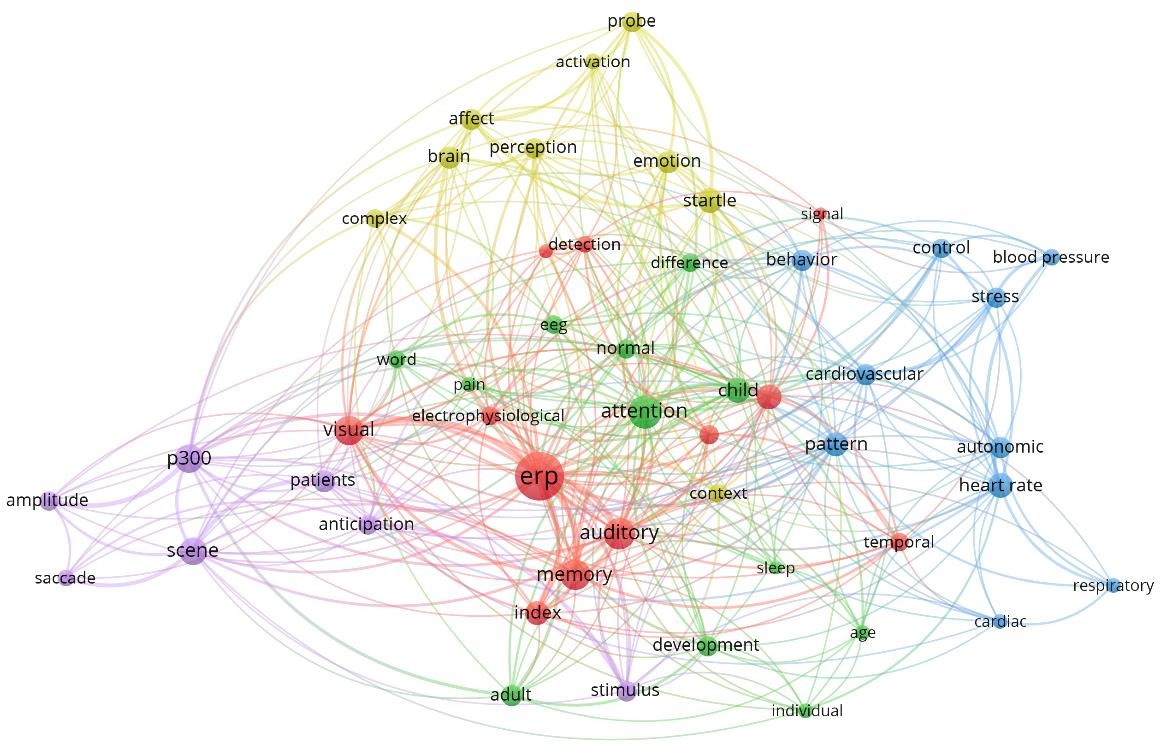
**

**Supplementary Figure 9.** Co-occurrence network for key terms extracted from publication titles (1994-2003).

**Supplementary Table 8.** Key terms and density for clusters of key term co-occurrence network (1994-2003).

| **Cluster** | **Key Terms** | **Size** | **Centrality** | **Density** |
| --- | --- | --- | --- | --- |
| 1 | erp, human, memory, visual, auditory, electrophysiological, index, method, mmn, signal, temporal, detection | 12 | 2261 (2) | 1699 (4) |
| 2 | attention, eeg, child, development, sleep, adult, difference, individual, word, age, normal, pain | 12 | 2297 (1) | 1937 (2) |
| 3 | cardiovascular, heart rate, autonomic, blood pressure, stress, pattern, behavior, control, cardiac, respiratory | 10 | 1668 (4) | 2068 (1) |
| 4 | startle, emotion, brain, affect, perception, context, probe, complex, activation | 9 | 2009 (3) | 1790 (3) |
| 5 | p300, scene, stimulus, patients, amplitude, anticipation, saccade | 7 | 1394 (5) | 1640 (5) |

***Note****. Key terms: key terms included in the cluster, sorted for occurrences. Size: number of key terms in the cluster. Centrality: Callon’s centrality as specified by Cobo et al. (2011) with cluster rank in parentheses. Density: Callon’s density as specified by Cobo et al. (2011) with cluster rank in parentheses.*


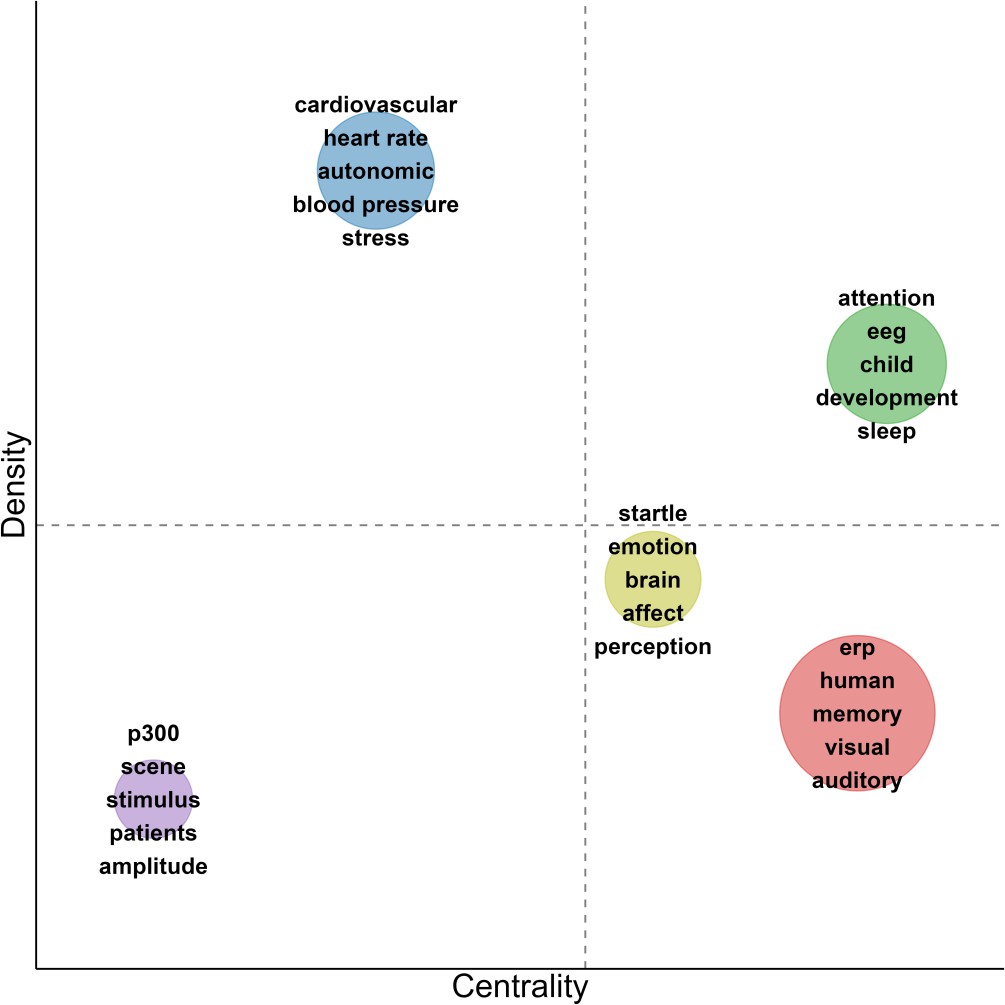


**Supplementary Figure 10.** Thematic map for key term clusters from 1994 to 2003. Each cluster is depicted as a circle with its size reflecting the total occurrences across all key terms in the cluster and its position indicating Callon’s centrality (x axis) and Callon’s density (y axis). Dashed lines indicate unweighted average centrality and density across all clusters. For each cluster, the 5 most frequent keywords are listed.

# Key Term Co-Occurrences (2004-2013)

**
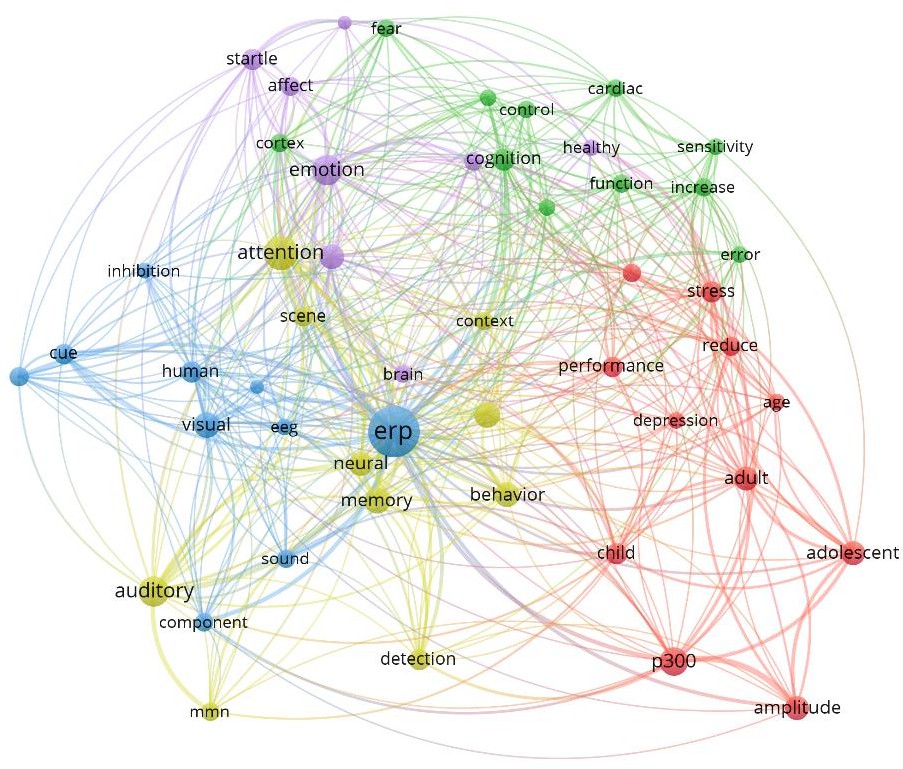
**

**Supplementary Figure 11.** Co-occurrence network for key terms extracted from publication titles (2004-2013).

**Supplementary Table 9.** Key terms and density for clusters of key term co-occurrence network (2004-2013).

| **Cluster** | **Key Terms** | **Size** | **Centrality** | **Density** |
| --- | --- | --- | --- | --- |
| 1 | cardiovascular, p300, stress, adult, amplitude, child, performance, adolescent, depression, reduce, age | 11 | 1809 (3) | 1994 (2) |
| 2 | cognition, error, function, increase, cardiac, time, cortex, mechanism, fear, sensitivity, control | 11 | 2003 (1) | 2134 (1) |
| 3 | erp, visual, eeg, human, cue, component, sound, activation, inhibition, paradigm | 10 | 1882 (2) | 1800 (4) |
| 4 | attention, electrophysiological, auditory, neural, memory, behavior, mmn, scene, detection, context | 10 | 1669 (5) | 1575 (5) |
| 5 | emotion, perception, startle, affect, brain, healthy, impact, prepulse inhibition | 8 | 1672 (4) | 1800 (3) |

***Note****. Key terms: key terms included in the cluster, sorted for occurrences. Size: number of key terms in the cluster. Centrality: Callon’s centrality as specified by Cobo et al. (2011) with cluster rank in parentheses. Density: Callon’s density as specified by Cobo et al. (2011) with cluster rank in parentheses.*


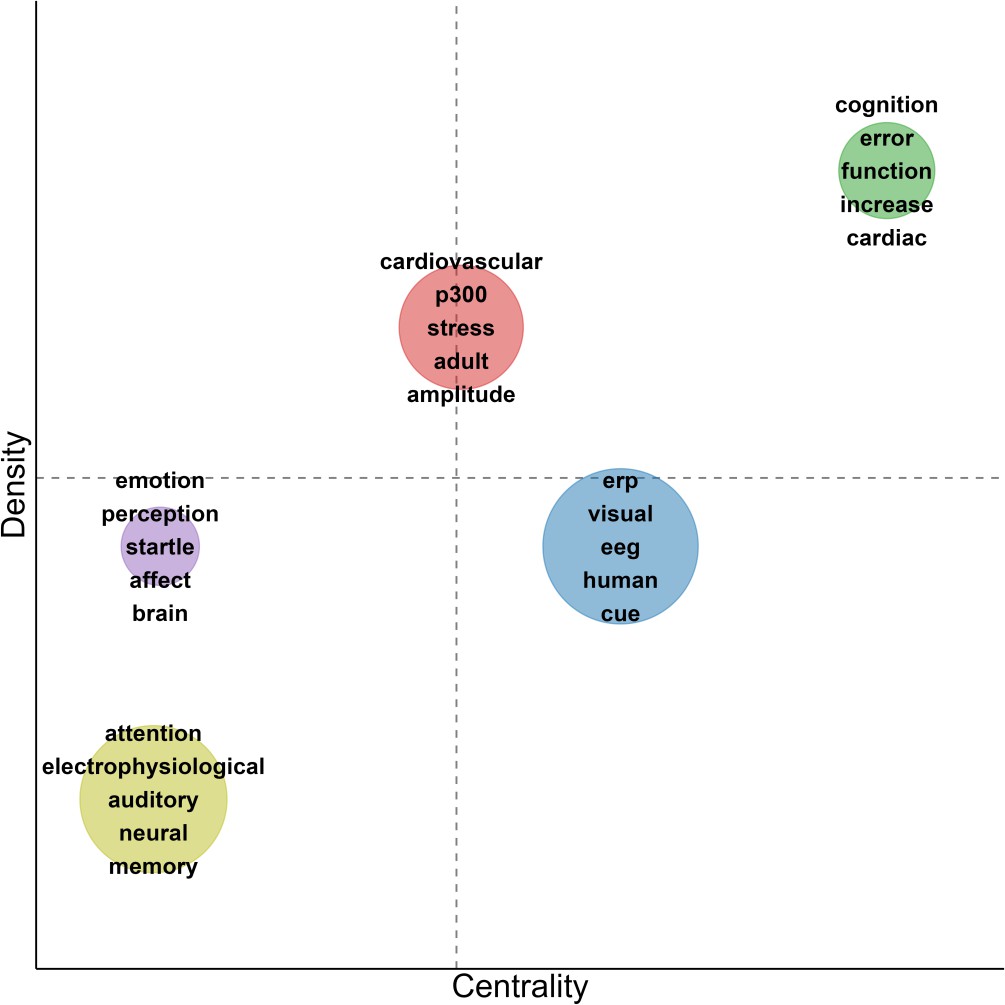


**Supplementary Figure 12.** Thematic map for key term clusters from 2004 to 2013. Each cluster is depicted as a circle with its size reflecting the total occurrences across all key terms in the cluster and its position indicating Callon’s centrality (x axis) and Callon’s density (y axis). Dashed lines indicate unweighted average centrality and density across all clusters. For each cluster, the 5 most frequent keywords are listed.

# Key Term Co-Occurrences (2014-2023)

**
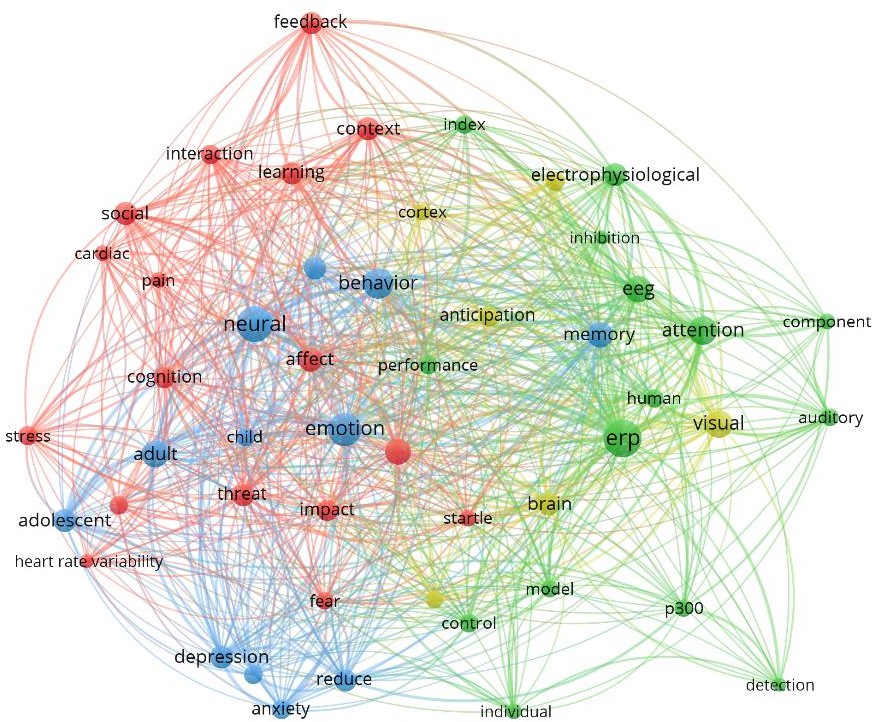
**

**Supplementary Figure 13.** Co-occurrence network for key terms extracted from publication titles (2014-2023).

**Supplementary Table 10.** Key terms and density for clusters of key term co-occurrence network (2014-2023).

| **Cluster** | **Key Terms** | **Size** | **Centrality** | **Density** |
| --- | --- | --- | --- | --- |
| 1 | perception, cognition, learning, affect, threat, stress, context, cardiovascular, social, pain, impact, feedback, fear, startle, cardiac, heart rate variability, interaction | 17 | 2529 (1) | 2088 (2) |
| 2 | erp, eeg, attention, electrophysiological, auditory, p300, human, model, component, inhibition, performance, detection, index, control, individual | 15 | 2188 (2) | 2182 (1) |
| 3 | emotion, neural, behavior, adult, memory, adolescent, depression, reduce, increase, reward, child, anxiety | 12 | 2139 (3) | 1499 (4) |
| 4 | visual, brain, dynamic, oscillations, anticipation, cortex | 6 | 1251 (4) | 1637 (3) |

***Note****. Key terms: key terms included in the cluster, sorted for occurrences. Size: number of key terms in the cluster. Centrality: Callon’s centrality as specified by Cobo et al. (2011) with cluster rank in parentheses. Density: Callon’s density as specified by Cobo et al. (2011) with cluster rank in parentheses.*


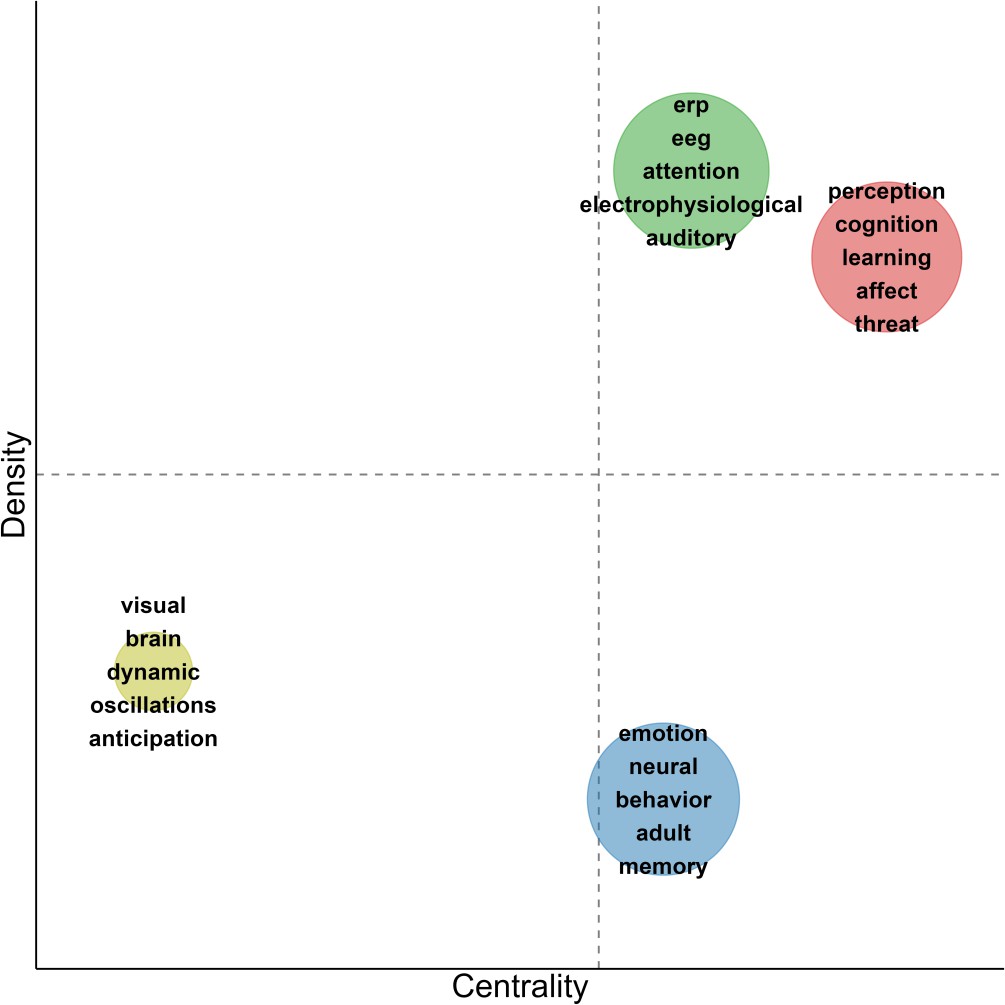


**Supplementary Figure 14.** Thematic map for key term clusters from 2014 to 2023. Each cluster is depicted as a circle with its size reflecting the total occurrences across all key terms in the cluster and its position indicating Callon’s centrality (x axis) and Callon’s density (y axis). Dashed lines indicate unweighted average centrality and density across all clusters. For each cluster, the 5 most frequent keywords are listed.
